# Cardiac amyloidosis detection from a single echocardiographic video clip: a novel artificial intelligence-based screening tool

**DOI:** 10.1093/eurheartj/ehaf387

**Published:** 2025-07-09

**Authors:** Jeremy A Slivnick, Will Hawkes, Jorge Oliveira, Gary Woodward, Ashley Akerman, Alberto Gomez, Izhan Hamza, Viral K Desai, Zachary Barrett-O’Keefe, Martha Grogan, Angela Dispenzieri, Christopher G Scott, Halley N Davison, Juan Cotella, Mathew Maurer, Stephen Helmke, Marielle Scherrer-Crosbie, Marwa Soltani, Akash Goyal, Karolina M Zareba, Richard K Cheng, James N Kirkpatrick, Tetsuji Kitano, Masaaki Takeuchi, Viviane Tiemi Hotta, Marcelo Luiz Campos Vieira, Pablo Elissamburu, Ricardo E Ronderos, Aldo Prado, Efstratios Koutroumpakis, Anita Deswal, Amit Pursnani, Nitasha Sarswat, Amit R Patel, Karima Addetia, Frederick L Ruberg, Michael Randazzo, Federico M Asch, Jamie O’Driscoll, Nora Al-Roub, Jordan B Strom, Liam Kidd, Sarah Cuddy, Ross Upton, Roberto M Lang, Patricia A Pellikka

**Affiliations:** University of Chicago, Chicago, IL, 5758 South Maryland Avenue, M.C. 9067, Chicago, IL 60637, USA; Ultromics, Ltd., Cascade Way, Oxford Business Park, Oxford, OX4 2SU, UK; Ultromics, Ltd., Cascade Way, Oxford Business Park, Oxford, OX4 2SU, UK; Ultromics, Ltd., Cascade Way, Oxford Business Park, Oxford, OX4 2SU, UK; Ultromics, Ltd., Cascade Way, Oxford Business Park, Oxford, OX4 2SU, UK; Ultromics, Ltd., Cascade Way, Oxford Business Park, Oxford, OX4 2SU, UK; Department of Cardiovascular Medicine, Mayo Clinic, 200 First Street SW, Rochester, MN 55905, USA; Department of Cardiovascular Medicine, Mayo Clinic, 200 First Street SW, Rochester, MN 55905, USA; Department of Cardiovascular Medicine, Mayo Clinic, 200 First Street SW, Rochester, MN 55905, USA; Department of Cardiovascular Medicine, Mayo Clinic, 200 First Street SW, Rochester, MN 55905, USA; Department of Cardiovascular Medicine, Mayo Clinic, 200 First Street SW, Rochester, MN 55905, USA; Department of Cardiovascular Medicine, Mayo Clinic, 200 First Street SW, Rochester, MN 55905, USA; Department of Cardiovascular Medicine, Mayo Clinic, 200 First Street SW, Rochester, MN 55905, USA; University of Chicago, Chicago, IL, 5758 South Maryland Avenue, M.C. 9067, Chicago, IL 60637, USA; Division of Cardiology, Columbia University, 622 West 168th Street, PH12-134, New York, NY 10032, USA; Division of Cardiology, Columbia University, 622 West 168th Street, PH12-134, New York, NY 10032, USA; Division of Cardiovascular Medicine, University of Pennsylvania, Hospital of the University of Pennsylvania, Room 11-131, 11th Floor South Pavilion, 3400 Civic Center Blvd., Philadelphia, PA 19104, USA; Division of Cardiovascular Medicine, University of Pennsylvania, Hospital of the University of Pennsylvania, Room 11-131, 11th Floor South Pavilion, 3400 Civic Center Blvd., Philadelphia, PA 19104, USA; Division of Cardiovascular Medicine, Ohio State University, 473 W. 12th Ave, Suite 200, Columbus, OH 43210, USA; Division of Cardiovascular Medicine, Ohio State University, 473 W. 12th Ave, Suite 200, Columbus, OH 43210, USA; Division of Cardiology, University of Washington, 1959 NE Pacific Street, Box 356422, Seattle, WA 98195-6422, USA; Division of Cardiology, University of Washington, 1959 NE Pacific Street, Box 356422, Seattle, WA 98195-6422, USA; Department of Cardiology, Hospital of University of Occupational and Environmental Health, 1-1 Iseigaoka, Yahatanishi-ku, Kitakyushu 807-8555, Japan; Department of Cardiology, Hospital of University of Occupational and Environmental Health, 1-1 Iseigaoka, Yahatanishi-ku, Kitakyushu 807-8555, Japan; Clinical Unity of Cardiomyopathies, Heart Institute (InCor), Av. Dr. Enéas Carvalho de Aguiar, 44, Cerqueira Cezar, Sao Paolo CEP: 05403/000, Brazil; Clinical Unity of Cardiomyopathies, Heart Institute (InCor), Av. Dr. Enéas Carvalho de Aguiar, 44, Cerqueira Cezar, Sao Paolo CEP: 05403/000, Brazil; Division of Cardiovascular Imaging, ICBA, Blanco Encalada 1543 street – C1428DCO, CABA, Argentina; Division of Cardiovascular Imaging, ICBA, Blanco Encalada 1543 street – C1428DCO, CABA, Argentina; Division of Cardiology, Centro Privado de Cardiología, Virgen De La Merced 550, Tucuman, Argentina; Department of Cardiology, University of Texas MD Anderson Cancer Center, 1515 Holcombe Blvd Unit 1451, Houston, TX 77030, USA; Department of Cardiology, University of Texas MD Anderson Cancer Center, 1515 Holcombe Blvd Unit 1451, Houston, TX 77030, USA; Division of Cardiology, Endeavor Health - NorthShore Cardiovascular Institute, Walgreen Building 3rd Floor, 2650 Ridge Ave, Evanston, IL 60201, USA; University of Chicago, Chicago, IL, 5758 South Maryland Avenue, M.C. 9067, Chicago, IL 60637, USA; Division of Cardiovascular Medicine, University of Virginia Medical Center, 1215 Lee Street, PO Box Charlottesville, VA 800158, USA; University of Chicago, Chicago, IL, 5758 South Maryland Avenue, M.C. 9067, Chicago, IL 60637, USA; Division of Cardiovascular Medicine, Boston University Chobanian & Avedisian School of Medicine, 821 Collamore Building, 72 East Concord Street, Boston, MA 02118-2308, USA; University of Chicago, Chicago, IL, 5758 South Maryland Avenue, M.C. 9067, Chicago, IL 60637, USA; Division of Cardiovascular Core Labs, MedStar Health Research Institute, 100 Irving St NW, Ste EB5123, Washington, DC 20010, USA; Diabetes Research Centre, College of Life Sciences, University of Leicester, Gwendolen Road, Leicester LE5 4PW, UK; Richard A. and Susan F. SmithCenter for Outcomes Research in Cardiology, MASCO 4375 Longwood Ave., Boston, MA 02215, USA; Richard A. and Susan F. SmithCenter for Outcomes Research in Cardiology, MASCO 4375 Longwood Ave., Boston, MA 02215, USA; Brigham & Women’s Hospital, 75 Francis Street, Boston, MA 02115, USA; Brigham & Women’s Hospital, 75 Francis Street, Boston, MA 02115, USA; Ultromics, Ltd., Cascade Way, Oxford Business Park, Oxford, OX4 2SU, UK; University of Chicago, Chicago, IL, 5758 South Maryland Avenue, M.C. 9067, Chicago, IL 60637, USA; Department of Cardiovascular Medicine, Mayo Clinic, 200 First Street SW, Rochester, MN 55905, USA

**Keywords:** Amyloid, Cardiomyopathy, Artificial intelligence, Echocardiography, Heart failure

## Abstract

**Background and Aims:**

Accurate differentiation of cardiac amyloidosis (CA) from phenotypic mimics remains challenging using current clinical and echocardiographic techniques. The accuracy of a novel artificial intelligence (AI) screening algorithm for echocardiography-based CA detection was assessed.

**Methods:**

Utilizing a multisite, multiethnic dataset (*n* = 2612, 52% CA), a convolutional neural network was trained to differentiate CA from phenotypic controls using transthoracic apical four-chamber video clips. External validation was conducted globally across 18 sites including 597 CA cases and 2122 controls. Classification accuracy was assessed on the entire external validation dataset, and subgroup analyses were performed both on technetium pyrophosphate scintigraphy referrals, and individuals matched for age, sex, and wall thickness. Model accuracy was also compared with the transthyretin CA score and the increased wall thickness score within a subset of older heart failure with preserved ejection fraction patients with increased wall thickness.

**Results:**

Cardiac amyloidosis patients and controls displayed similar age, sex, race, and comorbidities. After the removal of uncertain AI predictions (13%), model discrimination and classification were excellent for the entire external validation dataset [area under the receiver operating characteristic curve (AUROC) 0.93, sensitivity 85%, specificity 93%], irrespective of CA subtype (sensitivity: light-chain = 84%, wild-type transthyretin = 85%, and hereditary transthyretin = 86%). Performance was maintained in subgroup analysis in patients clinically referred for technetium pyrophosphate scintigraphy imaging (AUROC 0.86, sensitivity 77%, specificity 86%) and matched patients (AUROC 0.92, sensitivity 84%, specificity 91%). The AI model (AUROC 0.93) also outperformed transthyretin CA score (AUROC 0.73) and increased wall thickness (AUROC 0.80) scores.

**Conclusions:**

This AI screening model—using only an apical four-chamber view—effectively differentiated CA from other causes of increased left ventricular wall thickness.

## Structured Graphical Abstract

**Structured Graphical Abstract:**
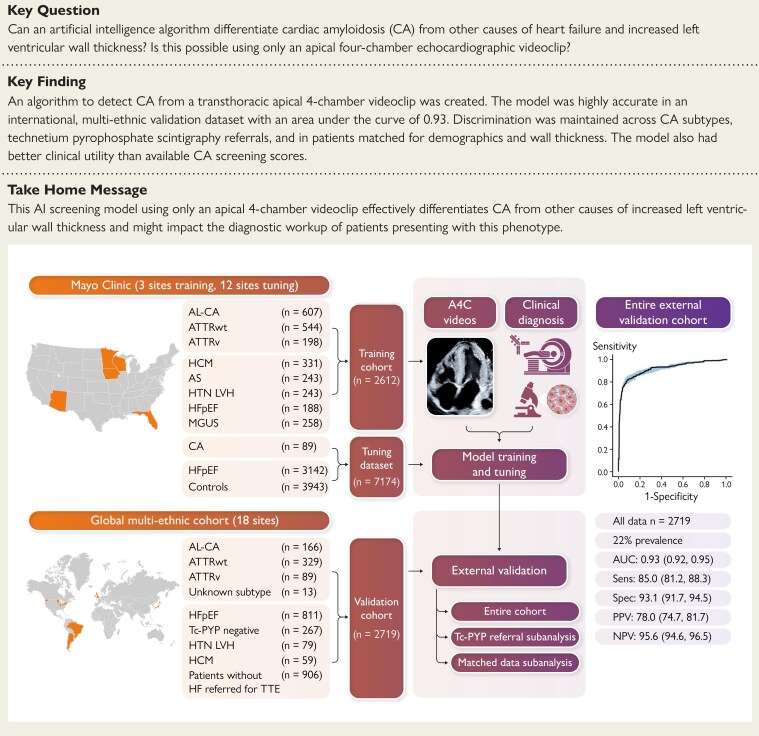
Geographic representation of the separate training, tuning, and international, multi-ethnic external validation cohorts which included patients with cardiac amyloidosis (CA) and controls referred for transthoracic echocardiography. The artificial intelligence model, based on a single apical four-chamber echocardiographic videoclip, was validated in the entire external validation cohort of 2719 patients, in which prevalence of CA was 22%, with AUC 0.93. The model's accuracy was maintained in testing in various subgroups.

**See the editorial comment for this article ‘AI-based screening of cardiac amyloidosis on standard echocardiography: spromising advances but need for real-world validation’, by A. Aimo *et al*., https://doi.org/10.1093/eurheartj/ehaf388.**

## Introduction

Cardiac amyloidosis (CA) is an infiltrative cardiomyopathy most commonly caused by deposition of abnormally folded transthyretin or immunoglobulin light chain proteins in the myocardium and is a frequent cause of heart failure with preserved ejection fraction (HFpEF). Given the recent availability of life-prolonging treatments for the two most prevalent types, it is critical that an accurate diagnosis be made to initiate treatment.^1,2^

Consensus guidelines recommend that patients with suspected CA undergo initial clinical evaluation, electrocardiography, and echocardiography.^3,4^ The presence of red flag features on these initial tests or elevation in CA-specific risk scores should then prompt further testing with cardiac magnetic resonance, technetium pyrophosphate scintigraphy (Tc-PYP), and/or invasive tissue biopsy.^4–8^ A diagnosis of CA is subsequently made by positive endomyocardial biopsy, positive extracardiac biopsy with typical cardiac imaging, or Tc-PYP features in the absence of a monoclonal light chain on comprehensive serum and urine analysis. Light chain biomarkers—highly sensitive for light chain dyscrasias—are critically important for excluding light-chain CA (AL-CA) prior to referral for Tc-PYP given the potential for false positive results in this subtype.^5^

Given its low cost and widespread availability, echocardiography is the initial imaging screening test of choice for suspected CA and plays a key gatekeeper role in this evaluation. However, recent studies have found that commonly recognized echocardiographic features of CA—such as increased relative wall thickness and abnormal global longitudinal strain with apical sparing—may be less sensitive and specific than initially presumed.^8–11^ Potential explanations for the latter include the phenotypic overlap with other diseases, coexistence of cardiac comorbidities, recent inclusion of less advanced stage CA, and more stringent and clinically relevant choice of controls than in early studies. Multiparametric echocardiography models have been developed to identify CA; however, these are time-consuming and require heightened provider suspicion.^6–8^ It is estimated that a large proportion of patients with CA may be undetected at the earlier stages of the disease, resulting in delays in appropriate diagnostic workup and treatment, and missing the opportunities to prevent decline in quality of life and impact survival.^1,12^ Additionally, low specificity of echocardiographic findings may also result in excessive downstream testing and unnecessary invasive biopsies.^13,14^ Improvement in the current paradigm for the echocardiographic detection of CA is needed.

The recent application of artificial intelligence (AI) has led to a cascade of advancements in the field of echocardiography and appears poised to revolutionize the field. Studies have demonstrated the potential for deep learning (DL) to accurately identify cardiac disease from routinely acquired echocardiographic video clips.^15–17^ Building on our experience with development of an AI model to detect HFpEF from a single apical four-chamber video clip, we hypothesized that a model for the detection of CA generalizable across different ages, sexes, ethnicities, and ultrasound vendors could be developed.^17^

In this large, multicentre, multivendor study, we therefore sought to (i) train an AI model to detect CA using echocardiography, (ii) validate model performance in a large multiethnic patient population with echocardiographic data from a variety of ultrasound vendors, and (iii) compare the accuracy of the AI model to an existing clinical risk model for diagnosing CA.

## Methods

### Data

This retrospective, multisite, multiethnic cohort study was approved by the institutional review boards of each participating institution; patients who provided informed consent for participation in research were included. Data were collected from patients’ echocardiograms, between 2010 and 2022 from Mayo Clinic Health System (Rochester, MN, Scottsdale, AZ, Jacksonville, FL, Red Wing, MN, Albert Lea, MN, Austin, MN, Mankato MN, La Crosse, WI and five community outreach centres in the USA), University of Chicago (IL, USA), Brigham and Women’s Hospital (MA, USA), Beth Israel Deaconess Medical Center (MA, USA), St. George’s University Hospitals, National Health Service Foundation Trust (UK), Ohio State University (OH, USA), Columbia University Irving Medical Center (NY, USA), Instituto do Coração-INCOR (Brazil), Hospital of University of Occupational and Environmental Health (Japan), and University of Washington (WA, USA). This study received proper ethical oversight at each of the participating institutions in accordance with their institutional standards. A waiver of informed consent was issued by each site’s institutional review board to allow for the retrospective use of deidentified transthoracic echocardiography (TTE) images and clinical data.

### Participants

#### Model training and tuning datasets

The echocardiographic databases of three sites from Mayo Clinic Health System (Rochester, MN; Scottsdale, AZ; Jacksonville, FL) were screened to identify amyloid cases and controls who had undergone comprehensive TTE for model training and tuning. Echocardiograms were performed by certified cardiac sonographers and interpreted by experienced Level 3 trained physicians. Patients were eligible for inclusion if they were 18 years or older. To avoid bias from image artefacts related to prosthetic material, patients with prior cardiac implantable electronic devices or cardiac surgery were excluded. Possible cases were stratified by clinical site, age, sex, race, history of atrial fibrillation, and year of echocardiogram (Supplementary  *Figure 1*). Eligible controls identified from the Mayo Clinic electronic health record were then 1:1 matched to CA patients within the same strata (see Supplementary data online, *Methods* for more details). Controls were included if they had a primary diagnosis of hypertrophic cardiomyopathy (HCM), aortic stenosis (aortic valve peak velocity ≥ 2 m/s or ≥mild by physician interpretation),^18^ HFpEF with left ventricular ejection fraction (LVEF) > 40%, hypertension with increased left ventricular mass index (LVMI ≥ 95 g/m^2^ for females or ≥115 for males g/m^2^),^18^ or multiple myeloma or monoclonal gammopathy of unknown significance (MGUS); Further information on data collection methods and target sample sizes are reported in the Supplemental Methods and Supplementary  *Table 1*.

The dataset used for internal validation and model tuning has been described.^17^ Briefly, the echocardiographic database of the Mayo Clinic sites was screened to identify patients with a clinical diagnosis of HFpEF between 2009 and 2020. Patients with HFpEF (clinical diagnosis of heart failure, preserved systolic function, and echocardiographic evidence of increased left ventricular filling pressure) were matched to patients without HFpEF (no clinical diagnosis of heart failure, preserved systolic function, and no echocardiographic evidence of increased ventricular filling pressure) based on sex and year of echocardiogram. This dataset was intended to identify a broad and heterogeneous screening population, identifying a group of unselected cases with CA at a valid prevalence. There was no patient overlap between the training and tuning datasets.

#### External validation cohort

The model was externally validated utilizing a global, multicentre, multiethnic registry composed of 597 patients with CA as well as 2122 controls composed of patients with HFpEF, hypertension with left ventricular hypertrophy—defined as posterior wall thickness > 12 mm—obstructive, and non-obstructive HCM, patients with clinical and/or echocardiographic suspicion of CA in whom the diagnosis was excluded by Tc-PYP and non-heart failure controls referred for clinically indicated TTE. Cardiac amyloidosis patients and controls were identified via a retrospective review of institutional databases. Patients with prior valve surgery were not excluded from the external validation set.

### Diagnosis of cardiac amyloidosis

For the identification of cases with CA, patient medical records were reviewed and the determination of a patient as a case was only confirmed if the necessary criteria described in the multisocietal guidelines were fulfilled.^5^ Full details on the ground truth criteria are reported in Supplementary data online, *Methods*. In all instances, the echocardiogram closest to CA diagnosis was used for analysis. According to study design, the echocardiograms of controls were obtained at the closest time point to diagnosis of their respective condition or at the time of referral for Tc-PYP imaging. A clinical representative from each contributing site verified the ground truth criteria for each case before inclusion within the study.

### Overview of the artificial intelligence model

An ensemble of five 3D convolutional neural networks (CNNs) was developed on the training dataset using five-fold cross-validation (see Supplementary data online, *Methods* for details on model design and experiments on architecture and bench testing, Supplementary  *Tables 4–7*  & Supplementary  *Figures 2–5*). Using a prediction window of 30 frames, *n* minus 30 predictions were produced by each of the five models per clip, where *n* is the total number of frames per clip. Predictions from each model were averaged across time then averaged across the ensemble to obtain a single prediction probability for a clip. An ensemble of isotonic regression models was applied to the prediction score to obtain calibrated a prediction resembling a probabilistic estimate of CA. A binary prediction was then produced by thresholding the prediction probability at ≥0.06.

The model was also developed to provide non-diagnostic outputs for clips with high prediction uncertainty, where similar AI models have demonstrated a higher rate of false predictions.^17^ Model uncertainty was calculated using two measures: the maximum of the average prediction scores (uncertainty) and the standard deviation of the prediction scores across time (instability). Cut-offs for the model uncertainty parameters were chosen based on balancing optimum performance and subsequent rejection rates (see Supplementary data online, *Methods*). A clip was deemed uncertain for values above 0.32 and 0.22 for uncertainty and instability, respectively, which resulted in uncertain predictions in ∼11% of the tuning dataset. Thus, the AI model produced one of three possible outputs: (i) likely CA, (ii) unlikely CA, and (iii) no classification due to uncertainty.

### Comparison to clinical score

To compare the AI model against two commonly used clinical risk scores, both the transthyretin CA score (TCAS) and the increased wall thickness (IWT) score were calculated on a subset of the external validation dataset.^7,8^ Each of the clinical risk scores was implemented as reported in their initial studies (see Supplementary data online, *Methods* for details, Supplementary  *Tables 2 & 3*). To reflect the populations in which the TCAS and IWT score were validated, only those ≥60 years of age with clinical HFpEF and interventricular septum or posterior wall thickness ≥ 12mm—including transthyretin CA (ATTR-CA) and controls—were included in this subanalysis.^7,8^ Those with AL-CA, those with incomplete data for TCAS or IWT score calculation, and those in whom the AI model rendered an uncertain prediction were excluded. Scores ≥6, ≥8, and 0.06 were utilized for the TCAS, IWT, and AI models, respectively.

### Statistical analysis

Sample size considerations for the external testing dataset are described in Supplementary data online, *Methods* (*Supplementary Tables 8 & 9*). Briefly, a minimum sample size of 2625 was sought to detect a statistically significant difference between minimum performance criteria (derived from the literature; Supplementary data online, *Table S9*) and prototype performance using a binomial exact test and 90% power. Statistical optimization of the CNN was completed as described above. Measures of model calibration (slope, intercept, and the observed/expected ratio) discrimination [area under the receiver operating characteristic curve (AUROC)] and classification performance [sensitivity, specificity, positive predictive value (PPV), and negative predictive value (NPV)] were assessed during development and for the final model using bootstrapping, where 80% of the data were sampled 1000 times and data reported as the median (2.5th percentile, 97.5th percentile). Calibration statistics were evaluated from Loess smoothed flexible calibration curves, generated using the rms package in R Studio (v4.4.3). The Delong test was used to test for differences between AUROC estimates, and portion tests were used for sensitivity and specificity. The required alpha for a statistically significant difference was 0.05. Estimates of performance at different disease prevalences were also derived using the bootstrapping method where 80% of controls were randomly sampled and cases were then randomly sampled at the desired proportion. Continuous data are reported as median (interquartile range) (sample size). Missing clinical data were not imputed. Patients with missing image data (no available apical four-chamber view) were excluded. Analyses were performed using R (version 4.1) and Python (version 3.10). Within the subset of the external validation cohort in whom the TCAS and IWT score were calculated, decision curve analysis compared the AI model to the TCAS and IWT score to understand the model’s clinical utility for guiding referral to Tc-PYP in clinical practice, using decision thresholds from 0.1 to 0.35. Decision curves were modelled at a disease prevalence of 6.3%.^19^ The AI model, the TCAS, and the IWT score were modelled using their binary outputs, using cut-offs of ≥0.06, ≥6 and ≥8, respectively.

## Results

### Demographics

A consort diagram illustrating methods and diagnostic criteria for case and control selection in the internal training and tuning datasets is provided in Supplementary data online, *Figures S6–S18*. Ultimately, the training group included 1349 individuals with CA [607 (45.0%) AL-CA, 544 (40.3%) wild-type transthyretin CA (ATTRwt-CA), and 198 (14.7%) hereditary transthyretin CA (ATTRv-CA)] with 1263 controls with similar clinical characteristics of age, sex, race, history of arrhythmias, and year of echocardiographic acquisition. Controls included the following patients: 243 with aortic stenosis, 331 with HCM, 188 with HFpEF, 243 with hypertensive heart disease, and 258 with multiple myeloma or MGUS. The internal tuning dataset was composed of 7174 patients, including 3142 with HFpEF. Cardiac amyloidosis was present in 89 of these cases. The external validation dataset was composed of 597 CA patients (55% ATTRwt-CA, 28% AL-CA, 15% ATTRv-CA, and 2% other or unknown type) and 2122 controls; the control group was composed of 707 HFpEF, 267 with clinical suspicion of CA with negative Tc-PYP, 79 hypertensive patients with LVH, 59 HCM, and 1010 other patients referred for clinically indicated TTE (*Structured Graphical Abstract*). The demographics of the training, tuning, and external validation datasets are displayed in *Table 1*. A comparison of demographics in cases and controls within both the entire external validation dataset, Tc-PYP referrals, and age, sex, and wall thickness matched subgroups is presented in Supplementary data online, *Table S10*.

**Table 1:** Clinical and echocardiographic features of training, tuning, and external validation cohorts

Variable	Training *n* = 2612	Tuning *n* = 7666	External Validation *n* = 2719
Age, years, median (IQR)	70 (61, 77) [2611]	66 (54, 76) [7660]	71 (60, 80) [2397]
Body mass index, kg/m^2^, median(IQR)	27.6 (24.6, 31.5) [2608]	28.2 (22.4, 32.9) [7650]	27.6 (24.2, 32.0) [2281]
Male, *n* (%)	1916 (73.4)	3562 (46.5)	1524 (56.6)
Black, *n* (%)	328 (13.1)	75 (1.3)	522 (21.5)
Other race, *n* (%)	160 (6.4)	266 (4.6)	393 (16.2)
White, *n* (%)	2007 (80.4)	5422 (94.1)	1512 (62.3)
CA prevalence, *n* (%)	1349 (51.6)	89 (1.2)	597 (22.0)
ATTRwt CA, *n* (%)	544 (40.3)	24 (27.0)	329 (55.1)
ATTRv CA, *n* (%)	198 (14.7)	5 (5.6)	89 (14.9)
AL CA, *n* (%)	607 (45.0)	60 (67.4)	166 (27.8)
Unknown/other CA, *n* (%)	0 (0)	0 (0)	13 (2.2)
Hypertension, *n* (%)	1068 (40.9)	4378 (57.2)	1618 (70.9)
Diabetes mellitus, *n* (%)	507 (19.4)	1984 (25.9)	694 (30.2)
Left ventricular ejection fraction, median (IQR)	60 (49, 65) [2599]	63 (59, 66) [7659]	61 (55, 66) [2412]
Interventricular septal thickness, mm, median (IQR)	14 (12, 17) [2531]	10 (9, 12) [7349]	13 (10, 15) [2181]
Left ventricular posterior wall thickness, mm, median (IQR)	13 (11, 15) [2532]	10 (9, 11) [7336]	11 (10, 14) [2518]
Left ventricular mass index, g/m^2^, median (IQR)	127.0 (103.0, 156.0) [2516]	90.0 (76.0, 108.0) [7305]	113.0 (90.0, 141.7) [1294]

Numbers presented in square brackets indicate the total number of available data points.

AL, light chain amyloidosis; ATTRwt, wild-type transthyretin amyloidosis; ATTRv, hereditary transthyretin amyloidosis; CA, cardiac amyloidosis; IQR, interquartile range.

### Model training and tuning: classification and discrimination

A diagram of the final AI model architecture is presented in Supplementary data online, *Figure S19*. Prior to the implementation of model uncertainty measures, model discrimination was high on the training [AUROC 0.981 (0.967, 0.996)] and tuning datasets [AUROC 0.928 (0.893, 0.960)]. Using a standard cut-off of 0.5 for the prediction class probability, model classification accuracy in the training dataset, without uncertainty rejections, included sensitivity: 91.2% (86.8%, 97.7%), specificity: 96.2% (94.0%, 97.9%), PPV: 96.8% (94.9%, 98.2%), and NPV: 89.7 (85.3%, 97.2%). Within the tuning dataset—in which the prevalence of CA was 1%— model performance post-calibration using a cut-off of 0.06 was sensitivity: 89.0% (81.2%, 95.8%), specificity: 96.2% (95.6%, 96.6%), PPV: 24.0% (20.9%, 26.9%), and NPV: 99.8% (99.7%, 99.9%).

### External validation: discrimination, classification and calibration

Within the external validation dataset, we identified two key subgroups for additional analysis. A Tc-PYP referral subset consisted of 347 patients (23% CA) clinically suspected to have ATTR-CA and referred for a Tc-PYP scan. A matched subgroup consisted of 570 patients with 1:1 matching of CA cases with controls based on age, sex, and wall thickness. The purpose of these cohorts was to test the model in challenging situations such as (i) populations in which all patients were clinically suspected to have CA or (ii) those in whom controls were phenotypically similar.

**Figure 1:**
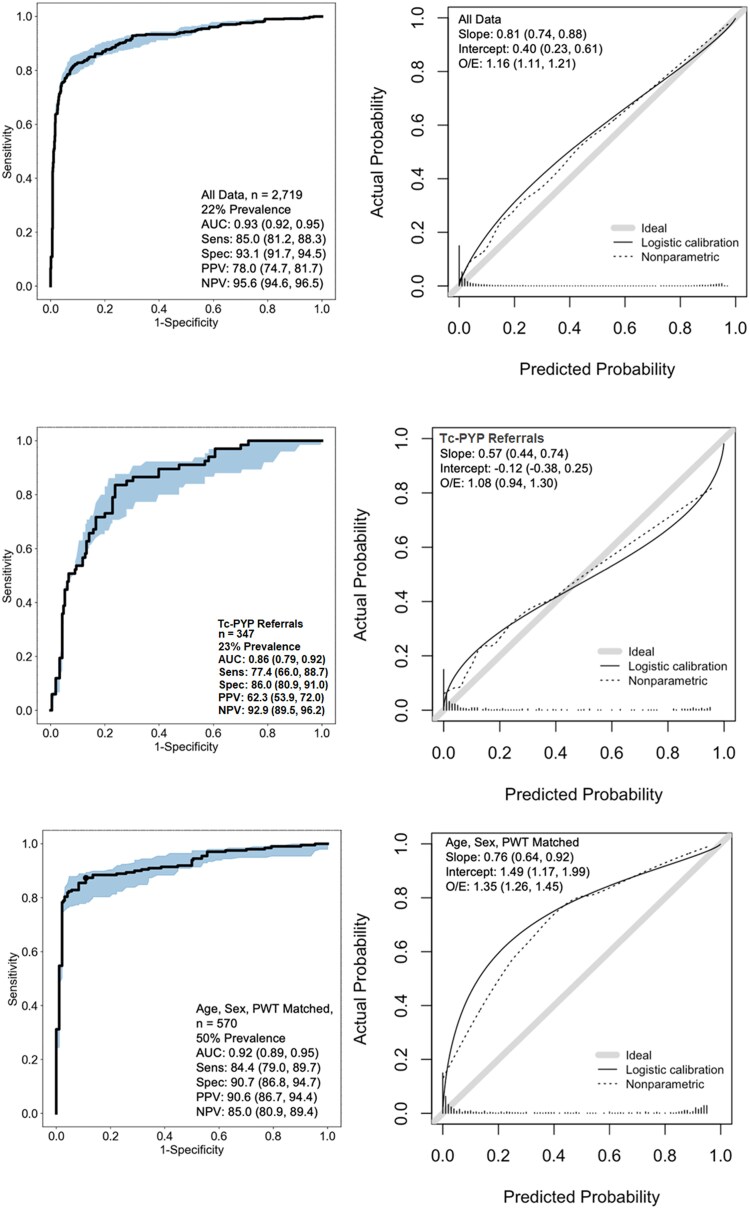
Discrimination, classification and calibration of the artificial intelligence model in independent testing datasets. Left-hand side: Receiver operating characteristic curves are depicted with 95% confidence intervals shaded. Statistics are presented on the dataset characteristics, including artificial intelligence discrimination and classification performance. Right-hand side: Smoothed flexible calibration curves present the actual and artificial intelligence-predicted probability of disease. Histograms at the bottom of each plot indicate the distribution of artificial intelligence-predicted probabilities. Both logistic and non-parametric flexible calibration curves are presented to visualize the effect of the underlying data distribution. AUC, area under the curve; NPV, negative predictive value; PPV, positive predictive value; Sens, sensitivity; Spec, specificity; Tc-PYP, technetium pyrophosphate scintigraphy

From a total of 2719 patients, the AI model produced positive/negative predictions in 2356 (86.6%) and uncertain predictions in 363 (13.4%). In patients with certain predictions, model discrimination [AUROC 0.93 (0.92, 0.95)] and classification [sensitivity: 85.0% (81.2%, 88.3%], specificity: 93.1% (91.7%, 94.5%), PPV: 78.0% (74.7%, 81.7%), and NPV: 95.6% (94.6%, 96.5%)] were high. Bootstrapped PPV and NPV estimates of the AI model are plotted across a range of disease prevalence in Supplementary  *Figure 20*. Model calibration suggests that the AI-predicted probability of disease tended to underestimate the observed probability in the dataset, which was more apparent in the lower probability ranges (*Figure 1*). The performance of the AI model was consistent across CA types. Among the AL-CA, ATTRwt, and ATTRv subtypes, the classification of the AI model was consistent with a sensitivity of 84.4%, 85.3%, and 86.3%, respectively.

Based on the entire external testing cohort, a comparison of demographics between those with correct, incorrect, or uncertain predictions is presented in Supplementary data online, *Table S11*. Compared with controls with correct AI predictions, those with incorrect or uncertain predictions had a high proportion of black patients and IWT. On the other hand, cases of incorrect or uncertain predictions had fewer males, patients from other race groups, and less wall thickening. Sensitivity analysis of model classification on the external testing dataset stratified by clinical characteristics, however, demonstrated consistent performance of the AI model across a range of demographics, comorbidities, LVEF, wall thickness, ultrasound vendor, and ground truth method (*Table 2*).

**Table 2:** Sensitivity analysis of model classification in clinical variables

Variable	*n*	Prevalence (%)	Sensitivity	Specificity	PPV	NPV
Age category (years)						
>80	494	18.0	82.0 (74.1, 90.6)	92.2 (88.6, 94.8)	69.9 (57.9, 78.7)	96.0 (94.1, 98.0)
71–80	594	33.2	87.6 (82.1, 92.2)	94.9 (92.6, 97.0)	89.6 (85.3, 93.7)	93.9 (91.0, 96.3)
61–70	463	27.6	81.0 (72.9, 86.9)	92.3 (89.5, 95.3)	80.5 (72.2, 88.0)	92.7 (88.6, 95.2)
50–60	322	17.4	79.6 (70.3, 91.1)	95.0 (91.2, 97.5)	77.9 (62.6, 87.3)	95.7 (93.5, 98.1)
<50	220	5.5	92.6 (70.0, 100.0)	97.7 (95.2, 99.4)	70.0 (42.2, 90.9)	99.4 (98.2, 100.0)
BMI category (kg/m^2^)						
>35	307	8.1	61.1 (40.5, 85.7)	95.9 (93.2, 97.8)	56.3 (35.0, 73.8)	96.8 (94.3, 98.9)
30–35	396	13.1	71.6 (52.8, 82.4)	95.6 (93.1, 97.8)	70.9 (51.8, 85.9)	95.6 (93.0, 97.4)
25–30	680	20.7	86.4 (79.4, 92.0)	95.3 (92.9, 97.1)	82.7 (75.6, 89.2)	96.4 (94.5, 98.0)
18.5–25	565	26.0	90.5 (85.5, 94.9)	91.5 (88.9, 94.2)	78.9 (72.8, 84.7)	96.4 (94.3, 98.2)
<18.5	38	23.7	100.0 (100.0, 100.0)	76.0 (61.4, 93.7)	58.1 (29.2, 85.2)	100.0 (100.0, 100.0)
Sex						
Female	1004	10.6	79.3 (70.0, 86.7)	93.9 (92.5, 95.3)	60.3 (49.7, 68.0)	97.6 (96.3, 98.6)
Male	1329	31.5	86.8 (83.6, 90.8)	92.5 (90.7, 94.3)	84.3 (80.9, 87.6)	93.8 (92.2, 95.8)
Race						
White	1328	22.3	85.7 (81.7, 89.7)	94.8 (93.5, 96.1)	82.7 (78.1, 87.1)	95.8 (94.7, 97.3)
Other	349	22.9	80.5 (72.2, 89.9)	94.5 (91.1, 97.3)	81.2 (72.1, 89.9)	94.4 (91.5, 97.0)
Black	419	32.7	88.4 (82.5, 92.6)	85.2 (80.5, 89.8)	74.3 (67.8, 80.8)	93.9 (90.8, 96.1)
Hypertension						
Y	1385	14.7	84.1 (77.9, 89.6)	93.7 (92.3, 95.1)	70.5 (63.5, 76.8)	97.1 (96.0, 98.2)
N	594	16.3	83.9 (75.7, 91.8)	94.0 (92.4, 96.4)	73.4 (66.5, 82.3)	96.8 (95.3, 98.5)
Diabetes mellitus						
Y	593	11.8	82.9 (73.3, 91.1)	94.3 (91.9, 96.4)	65.2 (54.5, 76.3)	97.7 (96.1, 98.9)
N	1408	16.4	84.3 (79.7, 88.4)	93.5 (91.9, 95.1)	72.6 (66.8, 78.2)	96.8 (95.8, 97.7)
Left ventricular ejection fraction (%)						
>65	641	12.0	81.0 (71.1, 88.4)	95.9 (93.6, 97.3)	72.7 (60.1, 82.0)	97.4 (96.0, 98.5)
50–65	1193	21.0	86.1 (81.9, 90.9)	93.9 (91.9, 95.6)	79.0 (72.6, 84.5)	96.2 (94.7, 97.6)
40–50	153	62.1	86.3 (78.5, 92.0)	84.2 (74.7, 92.9)	90.1 (83.4, 95.5)	79.2 (69.2, 88.1)
<40	118	72.0	85.3 (78.5, 92.4)	88.7 (73.1, 96.6)	94.8 (88.7, 98.5)	70.1 (57.8, 84.0)
Left ventricular posterior wall thickness (mm)						
>16	284	61.6	93.0 (89.2, 96.2)	83.7 (76.5, 90.9)	90.5 (85.5, 94.6)	88.5 (82.4, 93.5)
14–16	290	46.2	89.3 (81.3, 93.8)	85.4 (78.8, 91.5)	84.2 (77.8, 90.3)	90.2 (84.4, 94.7)
12–14	423	24.1	82.2 (73.5, 91.8)	91.7 (87.9, 95.1)	75.4 (66.5, 85.7)	94.2 (91.5, 97.1)
10–12	639	11.3	78.2 (66.7, 88.0)	95.0 (93.6, 96.9)	66.4 (56.7, 78.0)	97.1 (95.6, 98.7)
8–10	441	6.6	52.8 (31.6, 76.9)	97.3 (95.7, 98.9)	56.8 (36.6, 80.0)	96.7 (95.3, 98.8)
6–8	113	6.2	75.0 (26.3, 100.0)	100.0 (100.0, 100.0)	100.0 (100.0, 100.0)	98.8 (94.7, 100.0)
<6	7	0.0	-	-	-	-
Interventricular septal thickness (mm)						
>16	418	56.5	92.4 (88.9, 96.2)	85.4 (79.0, 91.1)	89.2 (84.1, 93.0)	89.3 (84.4, 94.1)
14–16	333	36.6	84.8 (78.1, 91.5)	88.5 (84.1, 93.5)	80.8 (75.1, 87.8)	91.3 (86.9, 95.0)
12–14	379	25.6	85.7 (77.4, 92.9)	92.0 (88.0, 94.9)	78.2 (70.1, 85.6)	94.8 (91.2, 97.5)
10–12	425	8.9	60.0 (45.5, 76.1)	94.9 (92.3, 96.9)	53.8 (39.3, 67.7)	96.0 (93.5, 97.6)
8–10	250	5.6	57.7 (21.8, 90.3)	96.9 (94.7, 98.7)	50.0 (25.0, 80.0)	97.8 (94.9, 99.5)
6–8	71	11.3	75.0 (50.0, 100.0)	100.0 (100.0, 100.0)	100.0 (100.0, 100.0)	97.9 (91.1, 100.0)
<6	5	0.0	-	-	-	-
Left ventricular mass index (g/m^2^)						
>95	769	39.4	87.2 (83.4, 90.6)	89.8 (86.9, 92.2)	84.6 (80.6, 88.2)	91.7 (88.6, 94.0)
<95	337	20.8	75.2 (61.6, 84.3)	97.3 (95.0, 98.9)	88.1 (79.1, 94.7)	93.6 (90.0, 96.3)

BMI, body mass index; NPV, negative predictive value; PPV, positive predictive value.

In the Tc-PYP referral and matched subgroups, the AI model produced uncertain predictions in 58 (16.7%) and 77 (13.5%), respectively. Discrimination of the AI model remained high in the Tc-PYP referral cohort [AUROC 0.86 (0.79, 0.92)], albeit lower than that observed in the entire external validation cohort. Classification remained good in the Tc-PYP subgroup [sensitivity: 77.4% (66.0%, 88.7%), specificity: 86.0% (80.9%, 91.0%), PPV: 62.3% (53.9%, 72.0%), and NPV: 92.9% (89.5%, 96.2%)] and model calibration indicated under prediction of disease likelihood at lower probability scores and over prediction of disease likelihood at higher probability scores. In the subgroup matched on age, sex, and wall thickness, the AI model demonstrated high discrimination [AUROC 0.92 (0.89, 0.95)] and classification[(sensitivity: 84.4% (79.0%, 89.7%), specificity: 90.9% (86.8%, 94.7%), PPV: 90.6% (86.7%, 94.4%), and NPV: 85.0% (80.9%, 89.4%)] while model calibration indicated under prediction of disease likelihood.

### Comparison with the transthyretin cardiac amyloidosis score and increased wall thickness score

**Figure 2:**
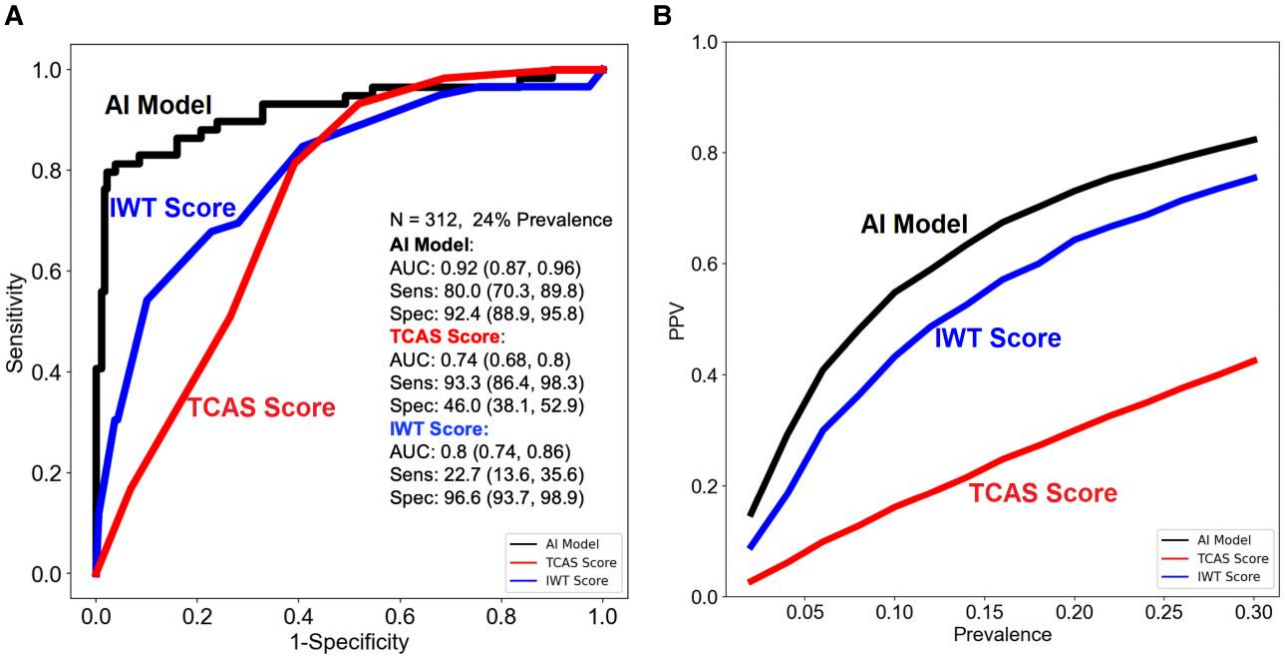
Discrimination and classification of the artificial intelligence model, transthyretin cardiac amyloidosis score, and increased wall thickness score. (*A*) Receiver operating characteristic curves for the artificial intelligence model, transthyretin cardiac amyloidosis score, and increased wall thickness score. Decision thresholds for the artificial intelligence model, the transthyretin cardiac amyloidosis score, and the increased wall thickness score were 0.06, 6, and 8, respectively. The reported statistics and confidence intervals represent the median, 2.5th and 97.5th percentiles from bootstrapping. (*B*) Positive predictive value of the artificial intelligence model, transthyretin cardiac amyloidosis score, and increased wall thickness score according to modelled disease prevalence. AI, artificial intelligence; AUC, area under the curve; IWT, increased wall thickness; NPV, negative predictive value; PPV, positive predictive value; Sens, sensitivity; Spec, specificity; TCAS, transthyretin cardiac amyloidosis score

From the external validation dataset, removal of those with AL-CA, under 60 years, with wall thickness < 12 mm, and without HFpEF resulted in subgroup of 955 patients. There were 312 patients with complete data for both the TCAS and IWT score and certain AI predictions. A description of clinical demographics of the CA and controls in the TCAS subgroup is provided in Supplementary data online, *Table S12*. Cases and controls had similar age and race, LVEF, and wall thickness. Comparing the performance of the AI model to the clinical scores, discrimination of the AI model [0.92 (0.87, 0.96)] was significantly higher than the TCAS [0.74 (0.68, 0.8), *P* < .001] and IWT [0.80 (0.74–0.86), *P* < .001] scores (*Figure 2A*). Sensitivity of the AI model [80.0% (70.3%, 89.8%)] was significantly lower than the TCAS [93.3% (86.4%, 98.3%) *P* = .02] and significantly higher than the IWT score [22.7% (13.6%, 35.6%), *P* < .001]. Specificity of the AI model [92.4% (88.9%, 95.8%)] was significantly higher than the TCAS [46.0% (38.1%, 52.9%), *P* < .001] and significantly lower than the IWT score [96.6% (93.7%, 98.9%), *P* = .04]. Comparison of the PPV at a disease prevalence of 1%–30% demonstrated consistently higher performance for AI compared with both the TCAS and IWT score (*Figure 2B*).

**Figure 3:**
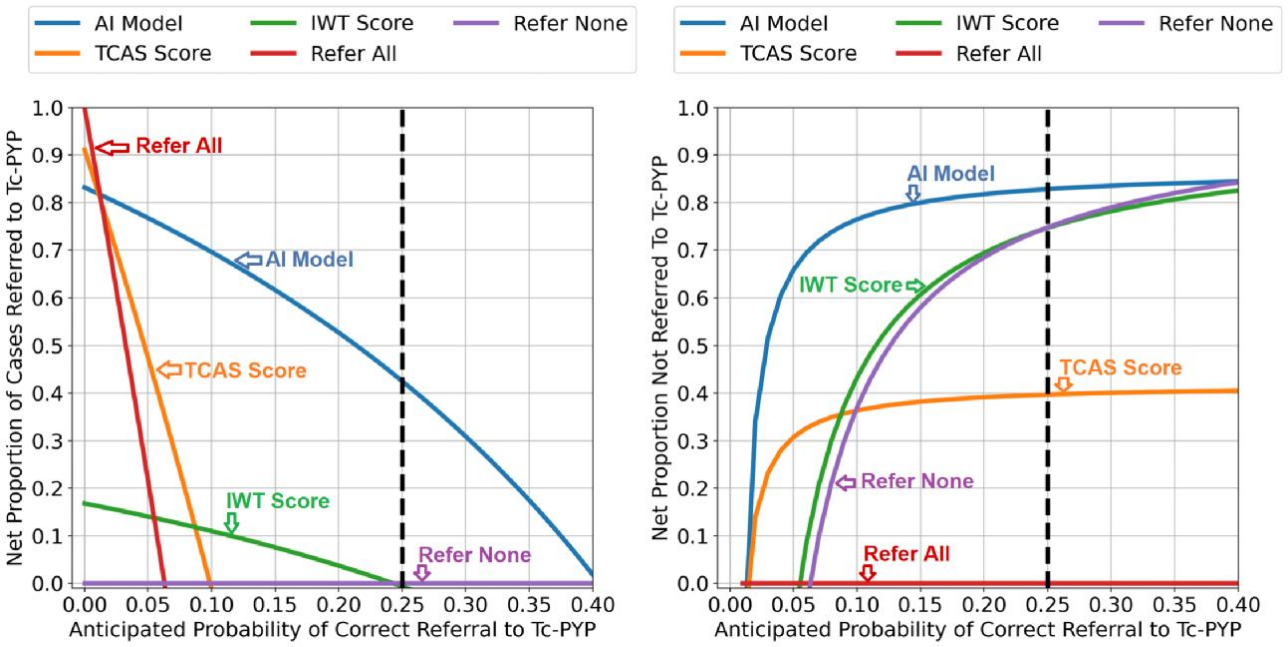
Decision curve analysis. Decision curve analysis comparing the net proportion of cases referred to technetium pyrophosphate scintigraphy (e.g. standardized net benefit) (left) and the net interventions avoided (right) for the artificial intelligence model (blue), the transthyretin cardiac amyloidosis score, the increased wall thickness score, referring all patients, and referring no patients. Disease prevalence has been modelled at 6.3%, as reported by AbouEzzeddine *et al*. The modelled clinical decision is whether to refer patients for Tc-PYP imaging, having ruled out positive light chains already, based on the output of the artificial intelligence model or clinical scores. The net proportion of cases referred to technetium pyrophosphate scintigraphy (standardized net benefit, left panel *y*-axis) reflects the net proportion of all cardiac amyloidosis patients who would be correctly referred with a given method, as a function of the decision threshold probability. Net interventions avoided describe the proportion of patients who would not be sent for unnecessary technetium pyrophosphate scintigraphy imaging using a given method, as a function of decision threshold probability. The decision threshold probability (*x*-axis) represents a patient’s/clinician's risk tolerance for undergoing technetium pyrophosphate scintigraphy imaging knowing the harms (unnecessary testing), benefits (diagnosing disease and access to treatment), and potential likelihood of a correct referral. Thus, the *x*-axis reflects the expected likelihood of a true positive referral (e.g. the dashed line at a decision threshold probability of 0.25 = one cardiac amyloidosis case found for every four referrals). Given the significant life-prolonging benefits of available therapies, a threshold of 0.25 was set for numerical reporting of this analysis, meaning that we would tolerate three incorrect technetium pyrophosphate scintigraphy referrals for every true cardiac amyloidosis patient identified by the tests in clinical practice. At this threshold, the artificial intelligence model, increased wall thickness, and transthyretin cardiac amyloidosis score identified a net proportion (e.g. without any false positives) 42.3%, 5.9%, and 0% of cases, indicating a 36.4% benefit of the artificial intelligence model over increased wall thickness. AI, artificial intelligence; IWT, increased wall thickness; TCAS, transthyretin cardiac amyloidosis score; Tc-PYP, technetium pyrophosphate scintigraphy

Decision curves evaluating the clinical utility of using the AI model or clinical scores for referring patients with suspected CA to Tc-PYP imaging are presented in *Figure 3*. Only the AI model had a net benefit for patients across the entire range of tested decision threshold probabilities. At the decision threshold probability of 0.25, constituting a scenario where clinicians are willing to refer four patients to Tc-PYP to find one case of CA, using the AI model would result in 36.4% more CA patients being correctly referred while reducing unnecessary referrals by 6.9%, compared with the next best method (IWT score). The TCAS and IWT score did not demonstrate a net benefit at this threshold.

## Discussion

In this study, we utilized a large, heterogeneous dataset to develop and externally validate a novel AI model. The model accurately differentiated CA from phenotypically relevant controls and generalized well across clinical sites, diverse patient populations, and ultrasound vendors. Furthermore, the AI model outperformed traditional echocardiography-based screening techniques, demonstrating a potential for reducing the false positive rate in low-prevalence (6.3%) screening while augmenting better detection of disease.

The AI model presented in this manuscript has the potential to improve both the accuracy and efficiency of CA detection compared with traditional TTE-based methods. Importantly, the model had sufficiently high PPV and NPV to demonstrate clinical utility, offering the potential to augment the frontline screening role that echocardiography plays in the evaluation of suspected CA. Furthermore, performance remained consistently high in those with clinical referrals to Tc-PYP and patients matched on age, sex, and wall thickness, indicating a low risk of false positives in phenotypically similar hearts without CA. A potential for false positive predictions at low prevalence remains, leaving a residual risk of unnecessary testing when employed in low-prevalence populations. Depending on the risk tolerance of patients and clinicians for unnecessary radiation exposure,^20^ and the perceived benefits or effectiveness of treatment, implementation of the AI model in screening could benefit patients. The decision curve analysis demonstrated that the AI model provides a net benefit (e.g. true-positive referrals for diagnostic testing exceed false positive), confirming that the benefits may outweigh the risks within most reasonable scenarios.

A strength of our study was the availability of a large population of highly phenotyped patients for model training and evaluation. This allowed for the development of a model using a dataset stratified on age, sex, ethnicity, and comorbidities. Furthermore, the large multicentre, multiethnic dataset for external testing afforded assessment of AI based on patient demographics, the degree of cardiac chamber remodelling, and ultrasound vendor. Nearly half of those in the validation cohort were non-White and a large percentage were African American, an ethnicity underrepresented in prior studies. The model remained accurate irrespective of age, sex, ethnicity, LVMI, LVEF, and left ventricular wall thickness. The generalizability of AI models across sexes and ethnicities is critical to ethical AI given the potential harm of prejudiced models towards underrepresented minorities.^21,22^ The model’s accuracy also remained similarly high across all ultrasound vendors and participating sites, further supporting its generalizability.

Our AI model also outperformed the accuracy of both the TCAS and IWT score in a subset of older adults with HFpEF and increased left ventricular wall thickness. These multiparametric risk scores were previously found to be accurate at differentiating ATTR-CA from phenotypically similar conditions including HFpEF and left ventricular hypertrophy.^7,8^ Decision curve analysis demonstrated that only the AI model had utility across a broad range of clinical scenarios, while the multiparametric scores demonstrated a net benefit only at lower risk thresholds, which may be clinically unjustifiable (e.g. 10 referrals to find one patient with CA). Overall, the TCAS and IWT score performed as originally reported, with high sensitivity or specificity, respectively, affording the opportunity to avoid missing cases or identify high-risk patients. The results from this study, however, demonstrate how the trade-off between sensitivity and specificity within low-prevalence scenarios risks poor clinical utility, potentially via excessive false positives or insufficient case detection. Thus, the ability of AI models to quantify and differentiate the complex features of disease with high sensitivity and specificity, coupled with resource-minimal clinical integration, has the potential to successfully augment the screening of rare and challenging diseases, beyond previously reported methods.

Our study aligns with a growing body of evidence supporting the role of AI to improve the echocardiographic detection of CA.^16^ Goto *et al*.^15^ developed an AI model derived from a single apical four-chamber view, which accurately differentiated CA from HCM, hypertension, and end-stage renal disease, irrespective of CA type. Limitations of that study are it did not report the race of participants and did not assess the performance of their model based on patient demographics or ultrasound vendor. Similarly, Duffy *et al*.^16^ developed a model derived from apical four-chamber views that differentiated CA from HCM and aortic stenosis cases; however, the AUROC in that study was lower for CA as compared with HCM, particularly in external validation. Similarly, this study did not compare the model accuracy based on age, sex, race, wall thickness, and/or ultrasound vendor. An additional strength of our study over the previous literature was the comparison of the model to the TCAS and IWT score, which demonstrated the superiority of our AI model over validated clinical risk scores.^7,8^ This expands on the current knowledge base by demonstrating the incremental value of DL over an existing risk prediction model. Nonetheless, these studies, as well as our own, demonstrate that AI can accurately differentiate CA from its phenotypic mimics using echocardiography.

Although we envision our model having broad applications to improve the echocardiographic detection of CA, there is a need to understand how this model will be integrated into the existing paradigm for CA diagnosis. Invariably, users will require training to understand the significance of the model’s output and how it should be incorporated into clinical decision-making. Additional processes may also need consideration to address questions and ethical concerns related to the sharing of patient images with companies for AI analysis. Prospective, randomized controlled trials and clinical implementation studies^23^ are also required to systematically study the safety and ethical concerns of AI algorithms and identify the optimal populations for screening. This could include specific testing in populations of interest such as those with HFpEF, low-flow, low-gradient aortic stenosis, light-chain neoplasms, carpal tunnel syndrome, and lumbar spinal stenosis.^3^ Additional studies could also look to understand how this model compares with, and could be supplemented by, other existing diagnostic tools including those using AI.^19,24,25^

### Limitations

The retrospective nature of this study may introduce bias in the reported performance estimates, as the severity of disease/phenotypes in this study may not fully represent those observed within a screening context. Although the CA and control groups in the external validation cohort were relatively similar with respect to age, race, and comorbidities, there was a larger proportion of males in the CA group; additionally, left ventricular wall thickness was higher in the CA group. We attempted to address this with our pre-specified subgroup analysis of CA patients 1:1 matched to controls based on age, sex, and wall thickness and found similar accuracy of the AI model. Additionally, all controls were not systematically tested for CA, which may frequently occur in older adults with HFpEF and low-flow, low-gradient aortic stenosis.^19,26–29^ However, this has also been a consistent limitation of prior studies in HFpEF and CA, including clinical trials, and is not unique to this study.^15,16,30^ However, the medical records were thoroughly reviewed in both the derivation and validation cohorts to assess for and exclude controls in whom CA was ultimately diagnosed. Future prospective studies are warranted to assess the accuracy of AI models for CA detection in populations who have undergone systematic screening for CA with Tc-PYP and/or tissue biopsy. The majority of patients in this study were also identified from high-volume, tertiary academic medical centres with the potential for selection bias; further studies are needed to assess the accuracy of the model in less specialized centres. Additionally, we included controls with MGUS in the training dataset; it is possible that some of these controls may have had normal echocardiograms that could confound model performance. However, we chose to include such controls as AL-CA may occur in up to 10% of patients with plasma cell dyscrasias, and the exclusion of cardiac involvement in this context is therefore clinically relevant.^31^ Another limitation of the model was that it assigned uncertain predictions in 13.4% of cases (see Supplementary data online, *Table S13* for AI performance including uncertain predictions). However, we felt it was important not to provide predictions in cases of uncertainty as this could negatively impact clinical care. Although the model performed well even in patients with lower left ventricular mass, we did not specifically validate the model in patients with early CA. Further long-term studies are needed to understand the potential for early diagnosis and the subsequent prognostic impact of AI-aided diagnosis in CA.

## Conclusions

In this large, global, multiethnic study, the AI model accurately differentiated CA from phenotypically relevant controls, irrespective of age, sex, ethnicity, and ultrasound vendor, and outperformed traditional TTE-based screening methods. The use of this rapid, fully automated AI model has the potential to improve the accuracy and efficacy of echocardiographic CA detection, thereby facilitating access to life-prolonging therapies.

## Supplementary Material

ehaf387_Supplementary_Data

## Declarations

### Data Availability

The data underlying this article will be shared on reasonable request to the corresponding author with restrictions that maintain patient confidentiality.

### Pre-registered Clinical Trial Number

Not applicable. This study was not registered.
