# Supplementary material for: Cardiac amyloidosis detection from a single echocardiographic video clip: a novel artificial intelligence-based screening tool
Source: Eur Heart J. 2025 Jul 9;46(40):4090–101. doi: 10.1093/eurheartj/ehaf387 (PMC12539910; doi:10.1093/eurheartj/ehaf387)
Supplement: ehaf387_Supplementary_Data [file ehaf387_supplementary_data.docx]

**Supplemental Materials**

**Supplemental Methods**:

**Sampling of Cases and Controls for Model Training**

Firstly, all potential cases from each of the sites (Mayo Rochester, Scottsdale and Jacksonville) were pooled and split into the three CA subtypes (ATTRwt, ATTRv & AL). The target number of samples from each subgroup is shown in Supplementary Table 1. Since cardiac amyloidosis is a relatively rare disease, cases target sample sizes (and subsequent matching of controls) were determined from preliminary evaluation of available data in electronic medical records. Within each sub-group, the data was split into strata based upon age, sex (binary), race and presence of arrhythmias (binary). The strata for age were <50, 51-60, 61-70, 71-80, >80 years. Strata for race were White, Black and Other. Therefore, data were stratified into a total of 60 strata (5 x 2 x 2 x 3). Data were randomly sampled from each strata an equal number of times until the target sample size was reached. Sampling from each strata was conducted in a consecutive manner according to the diagram in Supplemental Figure 1. Should sampling possibilities have been exhausted within a strata, or if no data was returned for a given strata, the remaining data was sampled from available data in other strata with a bias towards less representative categories. For example, if white males with no history of arrythmia between 61-70 years formed the majority of cases, data were sampled from other available strata if possible.

For eligible controls, patients were first split into each of the disease subgroups and then split into the same strata as cases (including race). Patients were then matched and then matched on a 1:1 basis to cases within each stratum, based upon the year of echocardiogram (2010-2012, 2013-2015, 2016-2018, 2019-2022) and clinical site (Rochester, Scottsdale and Jacksonville). Each case could only be matched to one control.

**Supplementary Table 1:** Target Sample size for the subgroups identified for AI model training.

| Subgroup | Total | Rochester | Phoenix | Jacksonville |
| --- | --- | --- | --- | --- |
| ATTRwt | 645 | 387 | 129 | 129 |
| AL | 645 | 387 | 129 | 129 |
| ATTRv | 210 | 126 | 42 | 42 |
| Non-Obstructive HCM | 300 | 180 | 60 | 60 |
| Aortic Stenosis | 300 | 180 | 60 | 60 |
| Hypertension with increased LVMI | 300 | 180 | 60 | 60 |
| Obstructive HCM | 100 | 60 | 20 | 20 |
| HFpEF without evidence of the above | 200 | 120 | 40 | 40 |
| Monoclonal gammopathy or multiple myeloma patient without cardiac amyloidosis | 300 | 180 | 60 | 60 |

**Supplementary Figure 1.** Patient selection stratum for model training

**Calculation of the TCAS and IWT Scores**

*TCAS Score:*

The Transthyretin Cardiac Amyloidosis Score is a validated risk model for ATTR-CA detection which utilizes age, sex, hypertension, LVEF, posterior wall thickness (PWT), and relative wall thickness (RWT). RWT was calculated as the product of the inter-ventricular septum thickness and the left ventricular posterior wall thickness The TCAS score has a minimum score of -1 and a maximum score of 10. Scoring was conducted as follows, with a score of ≥6 used as the high-risk cut-off.

**Supplementary Table 2:** Parameter values for the TCAS Score

| **Parameter** | **Score** |
| --- | --- |
| Age |  |
| ≥60 and < 70 years | 2 |
| ≥70 and < 80 years | 3 |
| > 80 years | 4 |
| Male Sex | 1 |
| Left Ventricular Ejection Fraction < 60 % | 1 |
| Hypertension | -1 |
| Left Ventricular Posterior Wall Thickness ≥ 12mm | 1 |
| Relative Wall thickness ≥ 0.57 | 2 |

*IWT Score:*

The Increased Wall Thickness Score is a multiparametric model utilising echocardiographic metrics. The model parameters are RWT, E/e’, tricuspid annular systolic plane excursion (TAPSE) longitudinal strain from the apical four-chamber view (LS) and the ratio between the segmental longitudinal strain of the apical septum and the basal inferoseptum (SAB). The RWT was calculated as (2xPWT)/Left ventricular end-diastolic diameter. The IWT has a minimum score of 0 and a maximum score of 10. Scoring was conducted as follows, with a score of ≥8 indicating the high-risk threshold.

**Supplementary Table 3:** Parameter values for the IWT Score

| **Parameter** | **Score** |
| --- | --- |
| SAB > 2.9 | 3 |
| LS ≥ -13 | 1 |
| TAPSE ≤ 19 | 2 |
| E/e’ > 11 | 1 |
| RWT ≥ 0.6 | 3 |

**AI Model Design and Architecture**

The AI model was trained and evaluated using Python (version 3.7.7) and TensorFlow (version 2.8) on one server with 4 Nvidia Tesla A100-SXM4-40GB graphic processing units. Bench testing experiments informing model design and architecture are reported below. All available apical four-chamber (A4C) video clips were used for training and validation of the model. Each video clip was pre-processed by cropping to the ultrasound region and resizing it to 128x128 pixels. Then, the clip was divided into sequences of 30 frames and each sequence was linearly rescaled to have intensities between 0 and 1.

An ensemble of five 3D Convolutional Neural Networks (CNN) was utilised, containing multiple convolutional blocks, each composed of 2 convolutional layers with kernels of 1x3x3 and 3x1x1 with batch normalization and swish activation. Maximum pooling layers were applied between pairs of convolutional blocks with strides of 1x2x2 for the first layer and 2x2x2 for the remaining ones. The last convolutional block was followed by a global average pooling layer, 2 fully connected layers with a dropout layer between them (dropout probability = 0.5) and a softmax activation layer. A 3D architecture was selected to leverage information both on spatial dimensions (kernel 1x3x3) and on the time dimension (kernel 3x1x1).

The ensemble was trained using a stratified 5-fold cross-validation where each CNN was trained independently on a different data split. During training, one sequence of 30 frames was randomly sampled per video clip, then the sequences were pre-processed and augmented with spatial transformations: scaling (by a factor of 80-120%), rotation (±20°), translation (vertical and horizontal by ±10%) and horizontal flipping. Image augmentation was randomly performed, applying one or a combination of multiple augmentations. Training was performed with the Adam optimizer using a cross-entropy loss, a learning rate of 5*10-5, batch size of 16. Every model was trained for a maximum of 400 epochs, however, training could stop earlier if the validation loss reached a plateau and did not reduce by 0.01 after 30 epochs. Additionally, the learning rate was reduced by a factor of 0.5 when the validation loss did not improve for 5 epochs. The performance of the AI model during training was evaluated using the area under the receiver operating characteristic curve (ROC curve, AUROC).

The prediction on an unseen video clip was done by applying the ensemble model to all overlapping 30-frame sequences with a stride of 1 and averaging across sequences, producing 5 pairs of prediction scores all using a cut-off of 0.5 for the prediction class probability during training. An ensemble of isotonic regression models was applied to the prediction scores to obtain calibrated prediction scores resembling an estimate of the long-term risk of cardiac amyloidosis. To more closely reflect the intended use population of a screening tool, model calibration was conducted using a disease prevalence of 5%. The average of the ensemble scores were thresholded at 0.06 after bench testing experiments determined it to be the most suitable cut-off for the final the diagnostic prediction (see below for additional information on threshold selection).

**Experiments on Model Design and Architecture**

Many aspects of the AI model architecture and design were chosen during bench testing of various considerations during the development process. The CA classifier’s architecture was designed as a 3D Convolutional Neural Network variant of the VGG-Net^1^, with the input number of frames, image size (designated below as the number of frames x pixel number x pixel number), batch normalisation and 3D design evaluated with experimentation. Different configuration parameters were considered and are summarised in Supplementary Table 1.

**Supplementary Table 4.** The different model configurations evaluated during bench testing.

| **Architecture** | **Input size** | **Parameters** | **Other configuration** | **Referred to as** |
| --- | --- | --- | --- | --- |
| 2D+1D variant architecture of VGG-Net ^1^ | 30x128x128 | 3.8M | Batch Normalisation and 3D separable convolution layers | CNN-128 |
| 2D+1D variant architecture of VGG-Net | 30x192x192 | 3.8M | Batch Normalisation and 3D separable convolution layers | CNN-192 |
| 2D+1D variant architecture of VGG-Net | 30x256x256 | 3.8M | Batch Normalisation and 3D separable convolution layers | CNN-256 |
| 2D+1D variant architecture of VGG-Net | 40x192x192 | 3.8M | Batch Normalisation and 3D separable convolution layers | CNN-40 |
| 2D+1D variant architecture of VGG-Net | 30x192x192 | 3.8M | Instance Normalisation and 3D separable convolution layers | CNN-instance |
| 3D variant architecture of VGG-Net | 30x192x192 | 3.8M | Batch Normalisation and standard 3D convolution layers | CNN-3D |
| HFpEF classifier ^2^ | 30x256x256 | 650k | A 3D CNN used to predict HFpEF | CNN-HFpEF |

Model architecture selection was performed by using two types of metrics. Firstly, classification performance metrics: sensitivity, specificity, accuracy, positive predictive value (PPV), negative predictive value (NPV), area under the curve (AUC); and secondly, metrics measuring the quality of the calibration of the model predictions: the Brier score, expected calibration error (ECE) and maximum calibration error (MCE).

The trained models were evaluated on the 5-fold hold-out data and the tuning data. Both evaluations used a bootstrapping approach. In every one of the 100 iterations of the bootstrapping approach, 80% of cases and controls were randomly sampled with replacement from the tested data. All performance metrics were calculated by using the per-model average predictions over *N* 30. A standard classification threshold of 0.5 applied on the mean prediction score was used to distinguish controls from cases. The corresponding mean and 95% confidence intervals (2.5^th^ to 97.5^th^ percentiles) of the evaluated performance on the 5-fold hold-out training and tuning data are summarised in Supplementary Tables 5 and 6, respectively.

**Supplementary Table 5**. Performance of each model on the training hold-out dataset.

| **Metric** | **CNN-128** mean  (5%, 95%) | **CNN-192** mean  (5%, 95%) | **CNN-256** mean  (5%, 95%) | **CNN-40** mean  (5%, 95%) | **CNN-instance** mean  (5%, 95%) | **CNN-3D** mean  (5%, 95%) | **CNN-HFpEF** mean  (5%, 95%) |
| --- | --- | --- | --- | --- | --- | --- | --- |
| **Sensitivity** | 0.912  (0.868, 0.977) | 0.949  (0.931, 0.974) | 0.952  (0.924, 0.977) | 0.938  (0.904, 0.973) | 0.930  (0.905, 0.951) | 0.977  (0.952, 0.997) | 0.951  (0.911, 0.975) |
| **Specificity** | 0.962  (0.940, 0.979) | 0.981  (0.963, 0.993) | 0.968  (0.911, 0.997) | 0.974  (0.953, 0.989) | 0.967  (0.941, 0.990) | 0.987  (0.968, 0.999) | 0.979  (0.957, 0.993) |
| **Accuracy** | 0.934  (0.906, 0.976) | 0.963  (0.951, 0.974) | 0.959  (0.923, 0.984) | 0.954  (0.936, 0.969) | 0.946  (0.928, 0.965) | 0.982  (0.961, 0.998) | 0.963  (0.945, 0.979) |
| **PPV** | 0.968  (0.949, 0.982) | 0.985  (0.970, 0.994) | 0.974  (0.927, 0.998) | 0.979  (0.961, 0.991) | 0.973  (0.950, 0.992) | 0.990  (0.975, 0.999) | 0.983  (0.966, 0.995) |
| **NPV** | 0.897  (0.853, 0.972) | 0.938  (0.917, 0.969) | 0.941  (0.908, 0.971) | 0.926  (0.891, 0.966) | 0.916  (0.889, 0.940) | 0.971  (0.936, 0.996) | 0.941  (0.897, 0.968) |
| **AUC** | 0.981  (0.967, 0.996) | 0.994  (0.988, 0.997) | 0.991  (0.976, 1.000) | 0.990  (0.982, 0.996) | 0.987  (0.978, 0.995) | 0.998  (0.994, 1.000) | 0.994  (0.988, 0.998) |
| **Brier** | 0.055  (0.028, 0.072) | 0.034  (0.028, 0.041) | 0.038  (0.017, 0.065) | 0.040  (0.030, 0.053) | 0.045  (0.032, 0.057) | 0.020  (0.008, 0.034) | 0.040  (0.030, 0.048) |
| **ECE** | 0.070  (0.055, 0.084) | 0.074  (0.063, 0.083) | 0.076  (0.067, 0.089) | 0.071  (0.060, 0.082) | 0.067  (0.055, 0.079) | 0.068  (0.057, 0.081) | 0.087  (0.073, 0.104) |
| **MCE** | 0.244  (0.137, 0.330) | 0.305  (0.196, 0.434) | 0.292  (0.161, 0.441) | 0.290  (0.166, 0.435) | 0.263  (0.158, 0.371) | 0.332  (0.175, 0.482) | 0.314  (0.208, 0.448) |

Note: PPV: Positive predictive value, NPV: Negative predictive value, AUC: Area under the receiver operating characteristic curve, ECE: Expected calibration error, MCE: Maximum calibration error.

**Supplementary Table 6.** Performance of each model on the tuning dataset

| **Metric** | **CNN-128** mean  (5%, 95%) | **CNN-192** mean  (5%, 95%) | **CNN-256** mean  (5%, 95%) | **CNN-40** mean  (5%, 95%) | **CNN-instance** mean  (5%, 95%) | **CNN-3D** mean  (5%, 95%) | **CNN-HFpEF** mean  (5%, 95%) |
| --- | --- | --- | --- | --- | --- | --- | --- |
| **Sensitivity** | 0.814  (0.716, 0.901) | 0.856  (0.778, 0.926) | 0.842  (0.765, 0.914) | 0.842 (0.771, 0.914) | 0.854 (0.778, 0.926) | 0.861 (0.790, 0.932) | 0.880 (0.803, 0.951) |
| **Specificity** | 0.928  (0.914, 0.941) | 0.903  (0.886, 0.921) | 0.907  (0.892, 0.919) | 0.913 (0.893, 0.930) | 0.904 (0.889, 0.916) | 0.896 (0.880, 0.908) | 0.856 (0.803, 0.917) |
| **Accuracy** | 0.927  (0.913, 0.939) | 0.902  (0.886, 0.920) | 0.906  (0.892, 0.918) | 0.912 (0.893, 0.929) | 0.903 (0.889, 0.915) | 0.895 (0.880, 0.907) | 0.857 (0.805, 0.916) |
| **PPV** | 0.132  (0.114, 0.154) | 0.107  (0.090, 0.129) | 0.109  (0.092, 0.123) | 0.116 (0.095, 0.139) | 0.107 (0.092, 0.121) | 0.100 (0.087, 0.114) | 0.083 (0.057, 0.123) |
| **NPV** | 0.997  (0.996, 0.999) | 0.998  (0.997, 0.999) | 0.998  (0.996, 0.999) | 0.998 (0.996, 0.999) | 0.998 (0.997, 0.999) | 0.998 (0.997, 0.999) | 0.998 (0.997, 0.999) |
| **AUC** | 0.928  (0.893, 0.960) | 0.930  (0.896, 0.964) | 0.936  (0.902, 0.967) | 0.929 (0.891, 0.961) | 0.933 (0.898, 0.963) | 0.932 (0.894, 0.965) | 0.936 (0.904, 0.967) |
| **Brier** | 0.066  (0.060, 0.073) | 0.078  (0.067, 0.089) | 0.078  (0.067, 0.091) | 0.072 (0.061, 0.086) | 0.077 (0.070, 0.085) | 0.082 (0.074, 0.092) | 0.109 (0.071, 0.140) |
| **ECE** | 0.179  (0.171, 0.192) | 0.188  (0.171, 0.208) | 0.193  (0.166, 0.224) | 0.178 (0.167, 0.198) | 0.184 (0.173, 0.194) | 0.184 (0.169, 0.209) | 0.246 (0.179, 0.297) |
| **MCE** | 0.745  (0.677, 0.810) | 0.792  (0.740, 0.839) | 0.774  (0.711, 0.833) | 0.756 (0.709, 0.814) | 0.754  (0.700, 0.809) | 0.798 (0.749, 0.842) | 0.747 (0.645, 0.823) |

Note: PPV: Positive predictive value, NPV: Negative predictive value, AUC: Area under the receiver operating characteristic curve, ECE: Expected calibration error, MCE: Maximum calibration error.

All models achieved high performance on all metrics. Model CNN-128 was selected for further testing and development, given that it offered the highest accuracy and PPV on the tuning dataset, which had a very low disease prevalence, combined with a low Brier score, ECE and MCE.

**Calibration of the Prediction Class Probabilities**

The clip prediction scores from the kth classification model were post-processed to correct overpredictions or underpredictions and approximate a class probability score using a calibration function. The aim was to obtain a binary classifier such that the output function better approximates the case proportion to a low prevalence, as is likely in the intended use population.

The calibration function was modelled as an ensemble of M=50 Isotonic Regression (IR)^3^ functions. Calibration functions were fitted on datasets with 1%, 2.5%, 5%, 7.5% and 10% prevalence, as would be expected in different screening applications. The IR functions were fit on a calibration dataset that consisted of the predictions of the five classification models on their corresponding fold’s hold-out data. At each iteration, we fit an IR function on 80% of randomly sampled data, maintaining the disease prevalence at the desired level. Bootstrapping was repeated for 50 iterations, resulting in an ensemble of 50 IR functions. To identify which disease prevalence results in the best calibration function, calibrated models were tested on the tuning dataset with a disease prevalence of 1-8% (based on the maximum feasible range obtainable with the tuning dataset sample size). The success of the calibration function was evaluated using the observed:expected ratio, the calibration slope and the calibration intercept.

Calibrating the model with 5% disease prevalence was deemed to result in the best calibration statistics across the disease prevalences evaluated in the tuning dataset (Supplementary Figure 2).


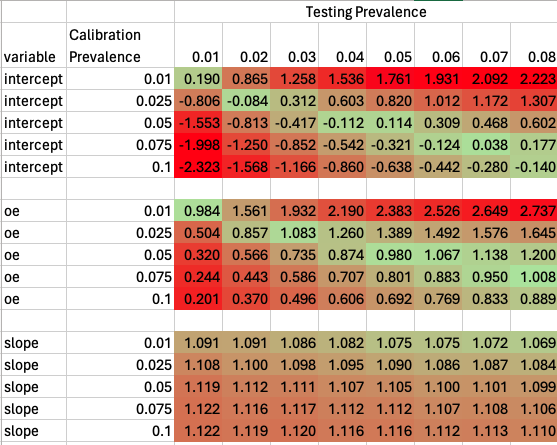


**Supplementary Figure 2**. Calibration performance of models fitted using a disease prevalence of 1-10% and tested on the tuning dataset at a disease prevalence of 1-8%.

**Selection of the Prediction Class Probability Cut-off for Binary Predictions**

Following the calibration of the class probability score, it was necessary to select a new threshold to produce the binary class output from the prediction class probability produced by the AI model. Any viable thresholds should permit acceptable performance across an anticipated range of disease prevalence based on different screening paradigms. Estimations of the prevalence of CA range from 0.017%^4^ in the general population, 0.4% in unselected clinical populations^5^, 1.2% in patients undergoing clinically indicated echocardiograms^6^ and up to 21% in elderly individuals with a diagnosis of heart failure with preserved ejection fraction^7^. In the ideal device, discrimination performance would be acceptable across the spectrum of possible use cases. Device performance was thus evaluated at a disease prevalence from 1% to 20% using a bootstrapping approach with 500 iterations. To increase the validity of the data tested at a prevalence between 10-20%, only patients with HFpEF and interventricular septal wall thickness >12mm were evaluated. The minimum performance criteria were obtained through evaluation of the published literature (described in External Testing Dataset Sample size considerations) and are presented in Supplementary Table 7. Acceptable performance was defined by one-sided binomial exact tests, evaluating whether the 2.5^th^ percentile of the observed performance on the tuning dataset was greater than the minimum performance criteria for sensitivity, specificity, PPV and NPV. The performance from the 2.5^th^ percentile was used to identify a threshold that performs the best under the worst-case scenario and to account for potential overfitting of the AI model to data originating from sites the model was trained on. Threshold with performance statistics from the 2.5^th^ percentile that were significantly greater (*p*<0.05) than the minimum performance criteria were considered acceptable for use.

**Supplementary Table 7. Minimum performance criteria for the AI model during development**

| Disease Prevalence | Minimum ppv | Minimum npv | Minimum Sensitivity | Minimum Specificity |
| --- | --- | --- | --- | --- |
| 1% | 3.31% | 99.63% | 70.90% | 79.10% |
| 2% | 6.47% | 99.25% | 70.90% | 79.10% |
| 3% | 9.50% | 98.88% | 70.90% | 79.10% |
| 4% | 12.38% | 98.49% | 70.90% | 79.10% |
| 5% | 15.15% | 98.10% | 70.90% | 79.10% |
| 6% | 17.80% | 97.71% | 70.90% | 79.10% |
| 7% | 20.34% | 97.31% | 70.90% | 79.10% |
| 8% | 22.78% | 96.90% | 70.90% | 79.10% |
| 9% | 25.12% | 96.49% | 70.90% | 79.10% |
| 10% | 27.37% | 96.07% | 70.90% | 79.10% |
| 11% | 29.54% | 95.65% | 70.90% | 79.10% |
| 12% | 31.63% | 95.22% | 70.90% | 79.10% |
| 13% | 33.64% | 94.79% | 70.90% | 79.10% |
| 14% | 35.58% | 94.35% | 70.90% | 79.10% |
| 15% | 37.45% | 93.90% | 70.90% | 79.10% |
| 16% | 39.25% | 93.45% | 70.90% | 79.10% |
| 17% | 41.00% | 92.99% | 70.90% | 79.10% |
| 18% | 42.68% | 92.53% | 70.90% | 79.10% |
| 19% | 44.31% | 92.06% | 70.90% | 79.10% |
| 20% | 45.89% | 91.58% | 70.90% | 79.10% |

The performance of the AI model on the tuning dataset by disease prevalence is presented in Supplementary Figures 3 and 4. Binomial exact tests demonstrated that the 2.5^th^ percentiles of performance met the minimum criteria only at the threshold of 0.06 (*p*<0.05) across all tested prevalences. Thus, 0.06 was chosen as the threshold.

**Supplementary Figure 3.**
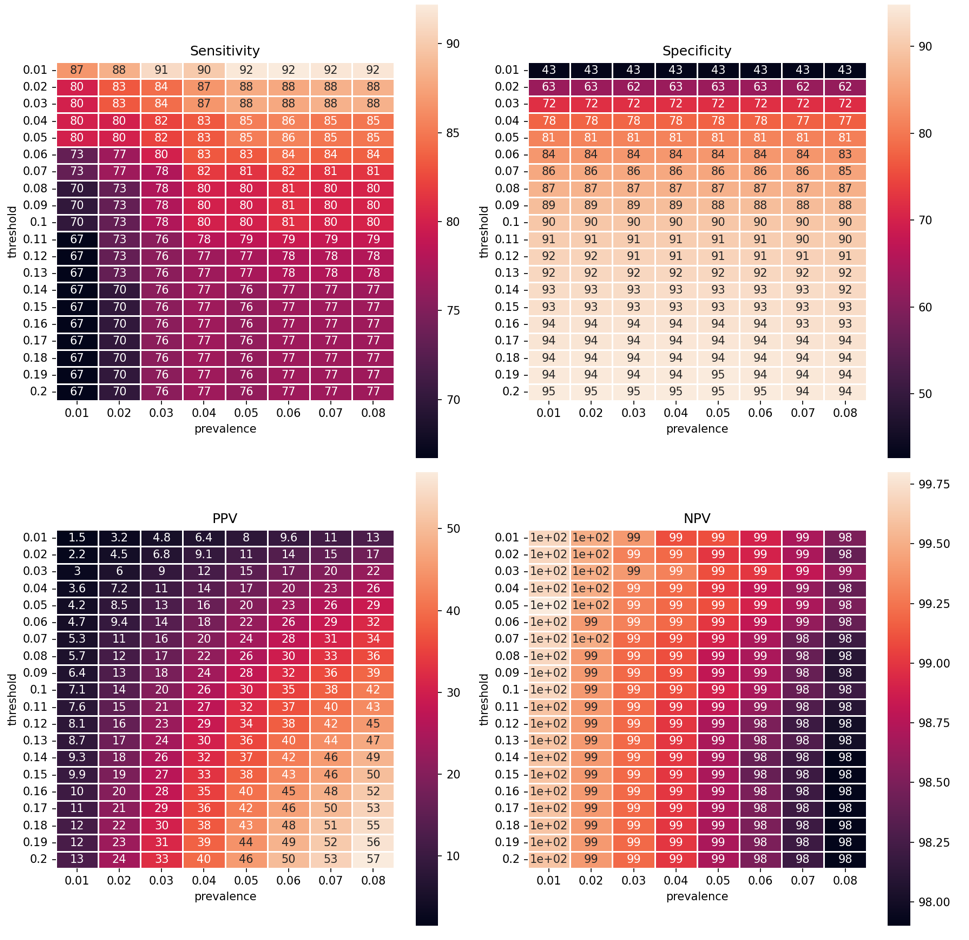
Performance of the AI model from the 2.5^th^ percentile of samples from the tuning dataset using binary thresholding from 0.01-0.08 (y-axis) at a disease prevalence of 1-8%.


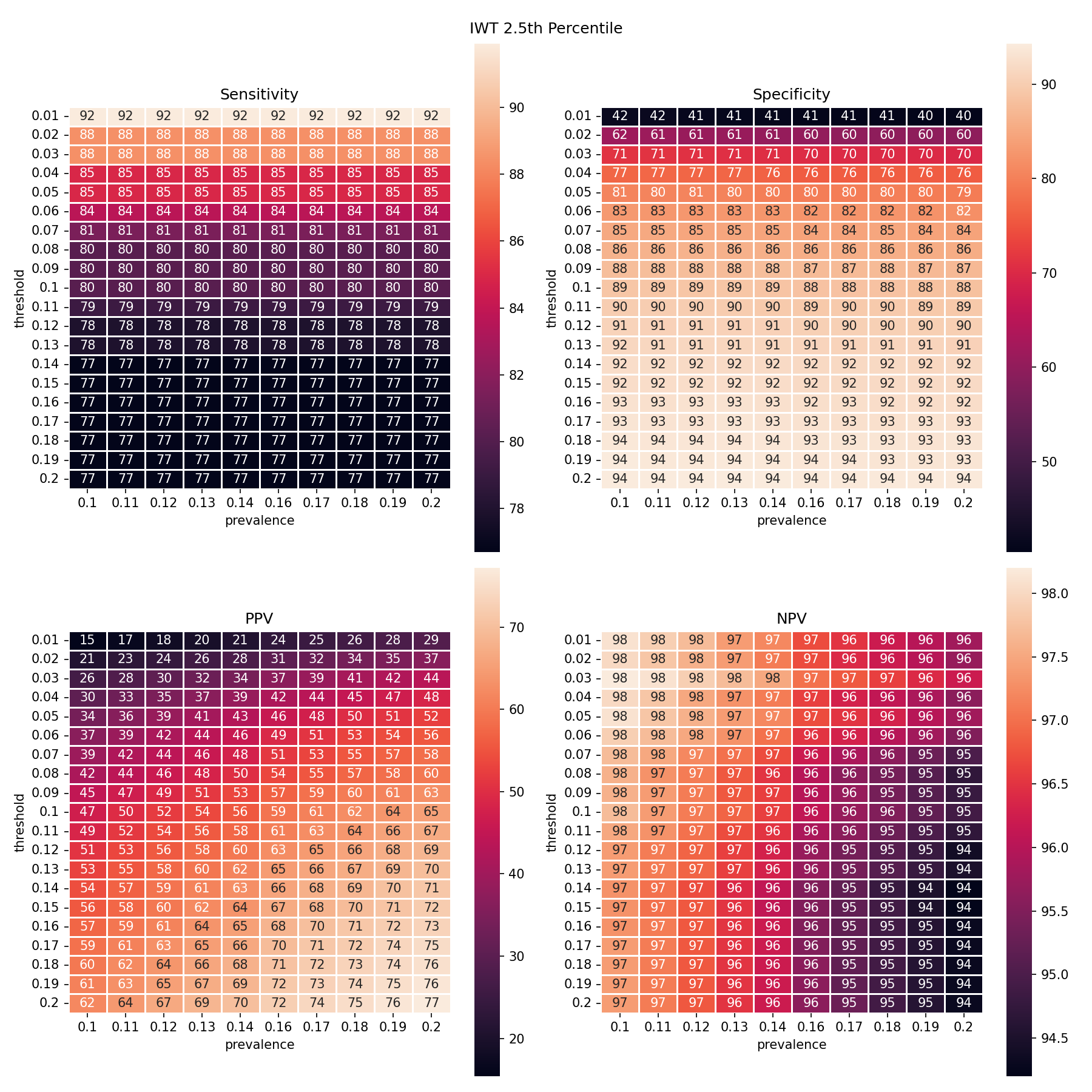


**Supplementary Figure 4.** Performance of the AI model from the 2.5^th^ percentile of samples from the tuning dataset using binary thresholding from 0.1-0.2 (y-axis) at a disease prevalence of 10-20%. Only patients with HFpEF and interventricular septal wall thickness >12mm were included.

**Selection of cut-offs for AI-model uncertainty**

Following the selection of the cut-off for binary classification for the calibrated model at 0.06, the identification of predictions with high uncertainty was then conducted to reduce the model error rate. Similar to the methods for a device detecting HFpEF from echocardiographic images^2^, we utilised the complement to maximum probability and the class probability standard deviation through time as measures of model uncertainty and instability, respectively. The classification model’s output is considered confident if the uncalibrated prediction score is close to 1 or 0; Conversely, the model’s output is less confident for scores near 0.5. As shown by Hendrycks & Gimpel^8^, correctly classified examples tend to have higher maximum probabilities than erroneously classified and out-of-distribution examples. Thus, for uncertainty, we utilised the complement to the max probability (1-max probability) of the mean clip prediction score. The instability (standard deviation) measures how dispersed the predictions are with respect to their mean and is also a common metric of uncertainty in Bayesian deep learning^9^.

To identify suitable cut-offs for the instability and uncertainty parameters, every possible combination over a reasonable range was tested and the resulting rejection rate, disease prevalence, sensitivity, and specificity were evaluated on the tuning dataset. Supplementary figure 5 shows how the rejection rate rapidly increased from 5-20% with relatively small changes in the parameters. Disease prevalence remained consistent in the tuning dataset regardless of the parameter threshold, suggesting that the proportion of rejected cases and controls was consistent regardless of the parameter cut-off. Evaluating the impact of different parameter combinations on classification performance demonstrated that sensitivity was largely unaffected while specificity tended to increase with increasing rejections, suggesting an uncertain high false positive rate. Performance improvements did not meaningfully increase above rejection rates of ~10% and so an instability threshold of 0.22 and an uncertainty threshold of 0.32 were selected to use in the model. This combination thus reduced the false positive rate by maintaining a high specificity of 96% and a sensitivity of 89%, with a rejection rate of 12%.


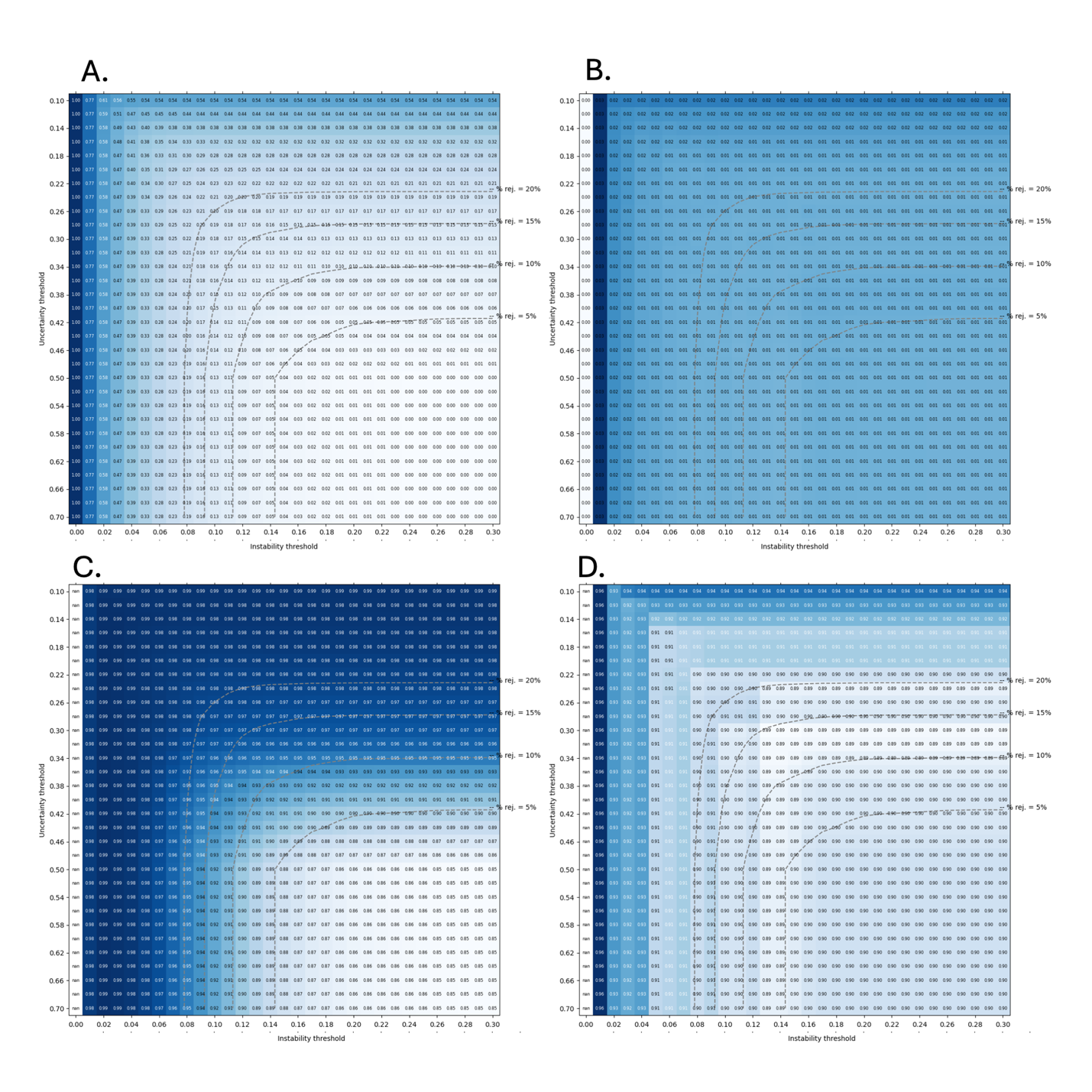


**Supplementary Figure 5.** Results of testing various thresholds for uncertainty and instability parameters. A) The rejection rate for each combination of parameters. B) Disease prevalence after rejection of uncertain studies according to each combination of parameters. C) Specificity of the AI model after rejection of uncertain studies according to each combination of parameters. D) Sensitivity of the AI model after rejection of uncertain studies according to each combination of parameters. Combinations of parameters that result in the rejection of 5%, 10%, 15% and 20% of studies are superimposed with dashed lines.

**Diagnosis of Cardiac Amyloidosis**

For the identification of cases with CA, patient medical records were reviewed and the determination of a patient as a case was only confirmed if the necessary criteria described in the multisocietal guidelines by Dorbala et al., were fulfilled^4^. Specifically, these criteria can be split into three categories: 1) histological diagnosis via endomyocardial biopsy, 2) histological diagnosis via extra cardiac biopsy and cardiac imaging features typical for CA or—in the case of AL-CA—elevated cardiac biomarkers 3) non-biopsy diagnosis of ATTR-CA via Tc-PYP, diphosphono-1,2-propanodicarboxylic acid  (DPD) or hydroxymethylene diphosphonate (HMDP) radiotracer uptake in the absence of a monoclonal protein on comprehensive light chains analysis. ATTR-CA was defined according to guidelines with amyloid detected by Congo Red staining and TTR confirmed as the precursor protein by either mass spectrometry or immunohistochemistry on endomyocardial biopsy or an extracardiac biopsy with typical cardiac imaging features, or a positive Tc-PyP in the absence of a monoclonal light chain on free light-chain assay or immunofixation electrophoresis of the serum and urine^4^. AL-CA was defined by the presence of positive endomyocardial biopsy or extracardiac biopsy with typical cardiac imaging features, and evidence of a monoclonal protein. In all instances, the echocardiogram closest to CA diagnosis was used for analysis. A clinical representative from each contributing site verified the ground truth criteria for each case before inclusion within the study.

**External Testing Dataset Sample size considerations**

For the training, the sample size was based on feasibility estimates after a review of the electronic medical records at The Mayo Clinic. Upon review of the database, it was considered that a target of 1,500 cases with cardiac amyloidosis was a reasonable expectation based on the amount of available data. The target sample size for controls was based on achieving a balanced dataset (n=1,500) while also achieving a balance between the control subgroups (e.g., HCM, HOCM, AS, HFpEF, MM). Target sample sizes are presented in Supplementary Table 8.

**Supplementary Table 8.** Target sample sizes for the training dataset.

| Subgroup | Total | Rochester | Scottsdale | Jacksonville |
| --- | --- | --- | --- | --- |
| ATTRwt | 645 | 387 | 129 | 129 |
| AL | 645 | 387 | 129 | 129 |
| ATTRv | 210 | 126 | 42 | 42 |
| Non-Obstructive HCM | 300 | 180 | 60 | 60 |
| Aortic Stenosis | 300 | 180 | 60 | 60 |
| Hypertension with increased LVMI | 300 | 180 | 60 | 60 |
| Obstructive HCM | 100 | 60 | 20 | 20 |
| HFpEF without evidence of the above | 200 | 120 | 40 | 40 |
| Monoclonal gammopathy or multiple myeloma without cardiac amyloidosis | 300 | 180 | 60 | 60 |

The tuning dataset sample size was also based on feasibility considerations. Through development of an AI model for the prediction of heart failure with preserved ejection fraction ^10^, data from Rochester, Jacksonville and Scottsdale were utilised. All available data was used for model tuning.

Sample size considerations for the external validation dataset were based on power calculations estimating the required number of patients to detect a difference with 97.5% confidence (1-alpha) and 80% power (1-beta) using the binomial exact test. The anticipated performance of the AI model was based on prototype performance (90% sensitivity and 83% specificity) which was to be compared against the clinical standard reported in the scientific literature. Studies were sought from the literature, published prior to November 2022) that reported the classification performance (sensitivity, specificity) of left ventricular regional strain, cardiac wall thickness and diastolic dysfunction; the three variables listed as typical imaging features in diagnostic guidelines ^11^. A total of 11 studies were included in the review. The true positive, true negative, false positive and false negative rates for each variable were entered into a meta-analytic analysis provided by Meta-DTA (<https://crsu.shinyapps.io/MetaDTA/>). Sensitivity and specificity estimates were weighted according to the reported sample size, and the average weighted performance was taken. Specifically, the minimum performance criteria for sensitivity was 70.9% and specificity was set at 79.1%. Using the minimum performance criteria for sensitivity and specificity, the minimum PPV and NVP were inferred using the following formulae^12^:

$$PPV= \frac{1}{1+exp(\emptyset1)\frac{1-\omega}{\omega}}$$

and

$$NPV= \frac{1}{1+exp(\emptyset2)\frac{1-\omega}{\omega}}$$

where

$$\emptyset1=\log\left( SP \right)-\log\left( SN \right)$$

$$\emptyset2=\log\left( SN \right)-\log\left( SP \right)$$

Where SN is the sensitivity, SP is the specificity and omega is the prevalence.

Thus, at a disease prevalence of 1.5%, acceptance criteria for PPV and NPV (as per the clinical standard) will be set at 4.9% and 99.4%, respectively.

**Supplementary Table 9.** Data used for determination of the minimum performance criteria.

| Author | Year | TP | FN | FP | TN | N | Sens | Spec | Weight_Sens | Weight_Spec |
| --- | --- | --- | --- | --- | --- | --- | --- | --- | --- | --- |
| Arana-Achaga et al ^13^ | 2023 | 68 | 40 | 89 | 29 | 226 | 0.63, | 0.246 | 2.817 | 2.874 |
| Boldrini et al ^14^ | 2020 | 433 | 214 | 76 | 255 | 978 | 0.669 | 0.77 | 2.907 | 2.979 |
| Boldrini et al ^14^ | 2020 | 239 | 93 | 22 | 150 | 504 | 0.72 | 0.872 | 2.865 | 2.912 |
| Boldrini et al ^14^ | 2020 | 252 | 80 | 28 | 144 | 504 | 0.759 | 0.837 | 2.876 | 2.9 |
| Boldrini et al ^14^ | 2020 | 282 | 50 | 43 | 129 | 504 | 0.849 | 0.75 | 2.89 | 2.846 |
| Boldrini et al^14^ | 2020 | 414 | 233 | 70 | 261 | 978 | 0.64 | 0.789 | 2.906 | 2.981 |
| Boldrini et al ^14^ | 2020 | 531 | 116 | 89 | 242 | 978 | 0.821 | 0.731 | 2.91 | 2.949 |
| Boldrini et al ^14^ | 2020 | 511 | 136 | 93 | 238 | 978 | 0.79 | 0.719 | 2.91 | 2.959 |
| Boldrini et al ^14^ | 2020 | 232 | 100 | 33 | 139 | 504 | 0.699 | 0.808 | 2.881 | 2.917 |
| Boldrini et al ^14^ | 2020 | 199 | 133 | 28 | 144 | 504 | 0.599 | 0.837 | 2.875 | 2.931 |
| Boldrini et al ^14^ | 2020 | 216 | 116 | 28 | 144 | 504 | 0.651 | 0.837 | 2.875 | 2.926 |
| Boldrini et al ^14^ | 2020 | 222 | 110 | 41 | 131 | 504 | 0.669 | 0.762 | 2.888 | 2.922 |
| Boldrini et al ^14^ | 2020 | 479 | 168 | 83 | 248 | 978 | 0.74 | 0.749 | 2.909 | 2.969 |
| Boldrini et al ^14^ | 2020 | 453 | 194 | 129 | 202 | 978 | 0.7 | 0.61 | 2.913 | 2.975 |
| Boldrini et al^14^ | 2020 | 466 | 181 | 132 | 199 | 978 | 0.72 | 0.601 | 2.913 | 2.972 |
| Boldrini et al^14^ | 2020 | 433 | 214 | 119 | 212 | 978 | 0.669 | 0.64 | 2.912 | 2.979 |
| Boldrini et al^14^ | 2020 | 209 | 123 | 22 | 150 | 504 | 0.63 | 0.872 | 2.864 | 2.928 |
| Boldrini et al^14^ | 2020 | 433 | 214 | 122 | 209 | 978 | 0.669 | 0.631 | 2.913 | 2.979 |
| Cuddy et al^15^ | 2022 | 240 | 84 | 99 | 175 | 598 | 0.741 | 0.639 | 2.896 | 2.954 |
| Cuddy et al^15^ | 2022 | 24 | 300 | 2 | 272 | 598 | 0.074 | 0.993 | 2.323 | 2.84 |
| Kyrouac et al^16^ | 2022 | 47 | 24 | 12 | 17 | 100 | 0.662 | 0.586 | 2.785 | 2.442 |
| Kyrouac et al^16^ | 2022 | 57 | 14 | 13 | 16 | 100 | 0.803 | 0.552 | 2.791 | 2.277 |
| Lofbacka et al^17^ | 2021 | 28 | 5 | 3 | 22 | 58 | 0.848 | 0.88 | 2.454 | 2.191 |
| Lofbacka et al l^17^ | 2021 | 28 | 5 | 7 | 18 | 58 | 0.848 | 0.72 | 2.598 | 2.149 |
| Nicol et al^18^ | 2020 | 60 | 22 | 1 | 31 | 114 | 0.732 | 0.969 | 2.564 | 2.456 |
| Nicol et al^18^ | 2020 | 65 | 17 | 11 | 21 | 114 | 0.793 | 0.656 | 2.797 | 2.356 |
| Nicol et al^18^ | 2020 | 63 | 19 | 5 | 27 | 114 | 0.768 | 0.844 | 2.731 | 2.406 |
| Nicol et al^18^ | 2020 | 78 | 4 | 8 | 24 | 114 | 0.951 | 0.75 | 2.786 | 1.889 |
| Pagourelias et al^19^ | 2017 | 19 | 21 | 8 | 52 | 100 | 0.475 | 0.867 | 2.464 | 2.756 |
| Pagourelias et al^19^ | 2017 | 15 | 25 | 4 | 56 | 100 | 0.375 | 0.933 | 2.216 | 2.749 |
| Pagourelias et al^19^ | 2017 | 20 | 20 | 0 | 60 | 100 | 0.5 | 1 | 1.71 | 2.757 |
| Pagourelias et al^19^ | 2017 | 35 | 5 | 17 | 43 | 100 | 0.875 | 0.717 | 2.652 | 2.551 |
| Phelan et al^20^ | 2012 | 51 | 4 | 5 | 25 | 85 | 0.927 | 0.833 | 2.675 | 2.049 |
| Robin et al^21^ | 2021 | 45 | 16 | 17 | 33 | 111 | 0.738 | 0.66 | 2.753 | 2.617 |
| Schiano-Lomoriello et al^22^ | 2016 | 21 | 12 | 0 | 60 | 93 | 0.636 | 1 | 1.671 | 2.745 |
| Schiano-Lomoriello et al ^22^ | 2016 | 23 | 10 | 10 | 50 | 93 | 0.697 | 0.833 | 2.475 | 2.711 |
| Usuku^23^ | 2023 | 27 | 5 | 20 | 11 | 63 | 0.844 | 0.355 | 2.636 | 2.207 |

Using the binomial exact test, binomial distributions about the expected (minimum performance criteria) and observed (anticipated based on the prototype)) proportions were estimated for increasing sample sizes to identify the minimum N required to detect a difference with 97.5% confidence (1-alpha) and 90% power (1-beta). The required power was set at 90% to inflate sample size estimates with the knowledge that performance estimates would be evaluated after the removal of “uncertain” predictions by the AI model and after potential removal of image studies that did not meet the inclusion criteria. Prototype performance estimates for sensitivity, specificity, PPV and NPV were 90%, 83%, 7.5% (1.5% prevalence) and 99.8% (1.5% prevalence), respectively. A lower limit prevalence of 1.5% was used as a representation of the lower limit to be expected within a screening population.

The minimum sample size was the lowest N whereby ≤90% of the distribution for estimated device performance falls below the 97.5th percentile (1-alpha) of the distribution for the acceptance criteria.

The “pbinom” function in R Studio was used to estimate the minimum sample size requirements, using the following code (with sensitivity as an example)

# input N

n = 47

# minimum performance criteria and anticipated performance, example for sensitivity

p0 = 0.709

p1 = 0.9

#calculate the minimum portion (e.g., critical value) at which 97.5% of the distribution for the acceptance criteria falls below

x=1:n

ic = (1-pbinom(x-1,n,p0)<0.025)

xm = min(x[ic])

# calculate what proportion of the observed distribution falls below the critical value, e.g., power

power = 1-pbinom(xm-1,n,p1)

Using this method, the minimum sample size required for each of the outcome measures was:

Sensitivity: 47

Specificity: 1,075

PPV: 887

NPV: 2,625

**Supplementary Results**

Consort Diagrams


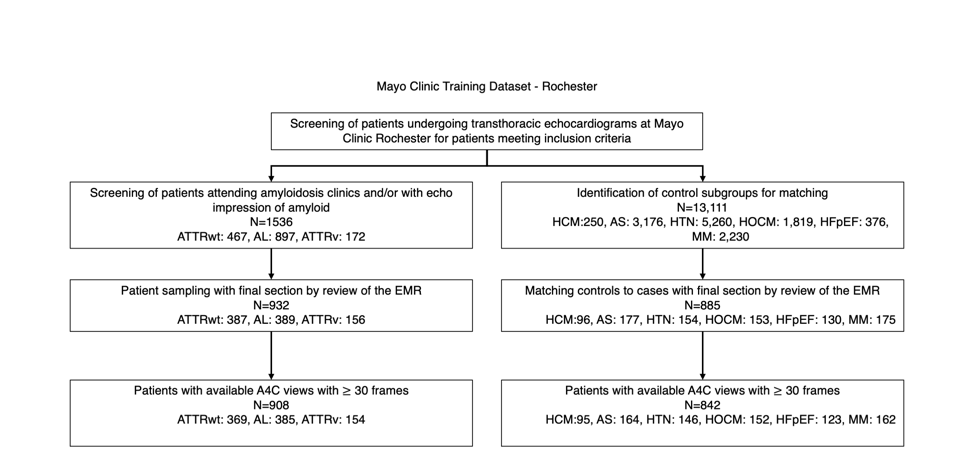


**Supplementary Figure 6.** Identification of patients from Mayo Clinic Rochester for device training and data flow data through the study.


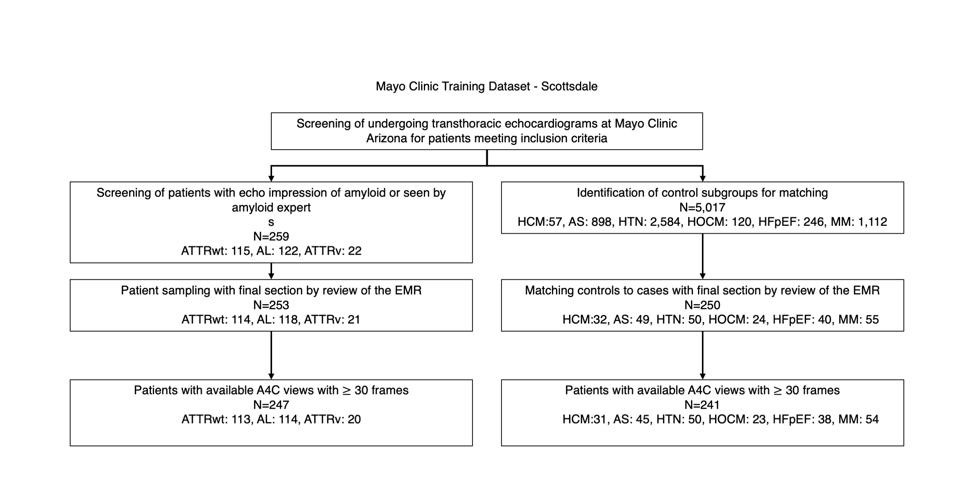


**Supplementary Figure 7.** Identification of patients from Mayo Clinic Scottsdale for device training and flow of data through the study.


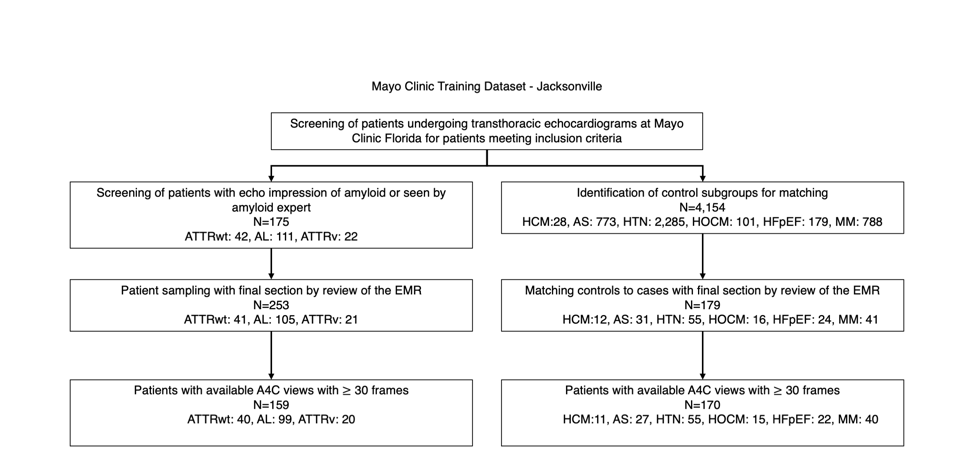


**Supplementary Figure 8.** Identification of patients from Mayo Clinic Jacksonville for device training and flow of data through the study.


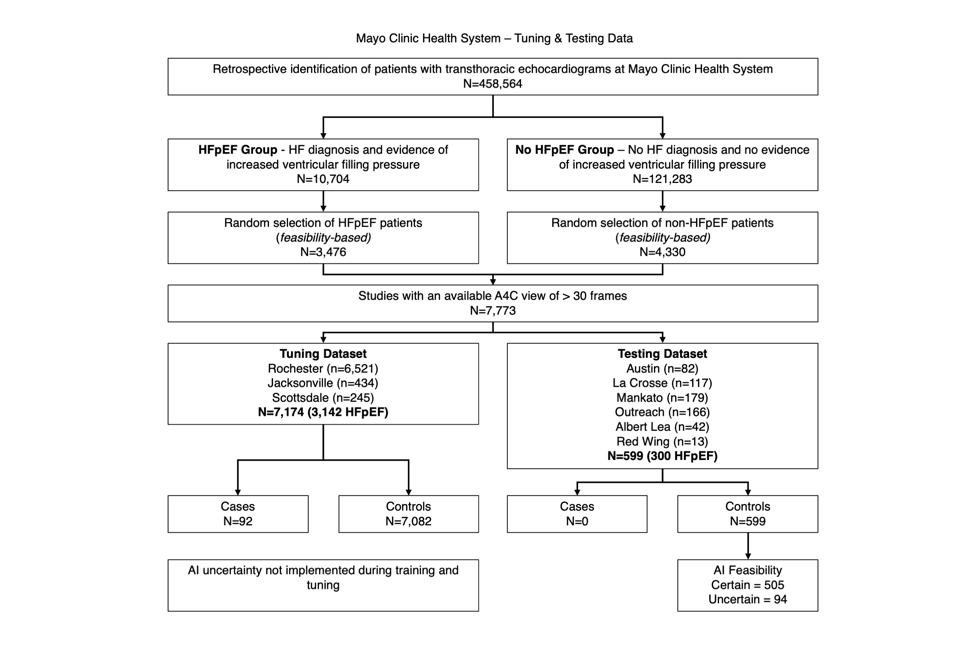


**Supplementary Figure 9.** Identification of patients from Mayo Clinic Health System for device tuning and external validation and subsequent flow of data through the study. “Feasibility based” is defined here as collecting the maximum number of available studies that project resources would allow.


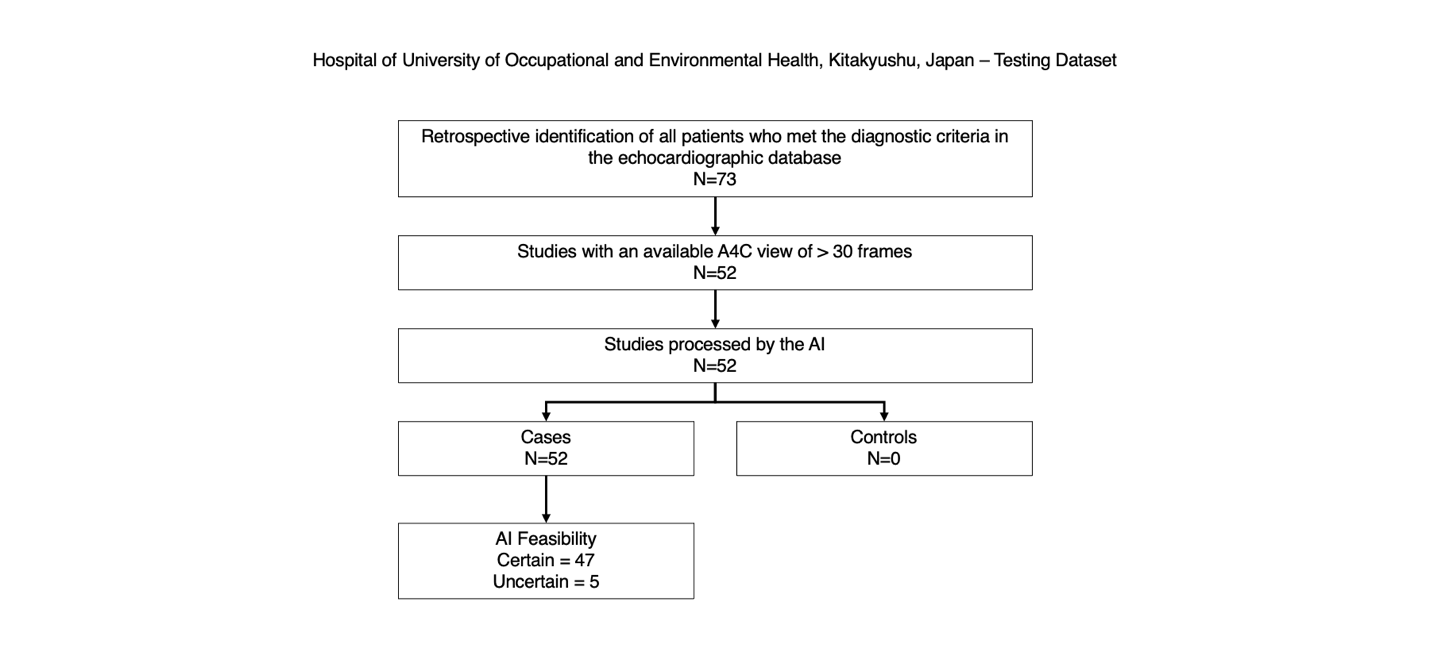


**Supplementary Figure 10.** Identification of patients from Hospital of University of Occupational and Environmental Health, Kitakyushu, Japan for external validation and subsequent flow of data through the study.


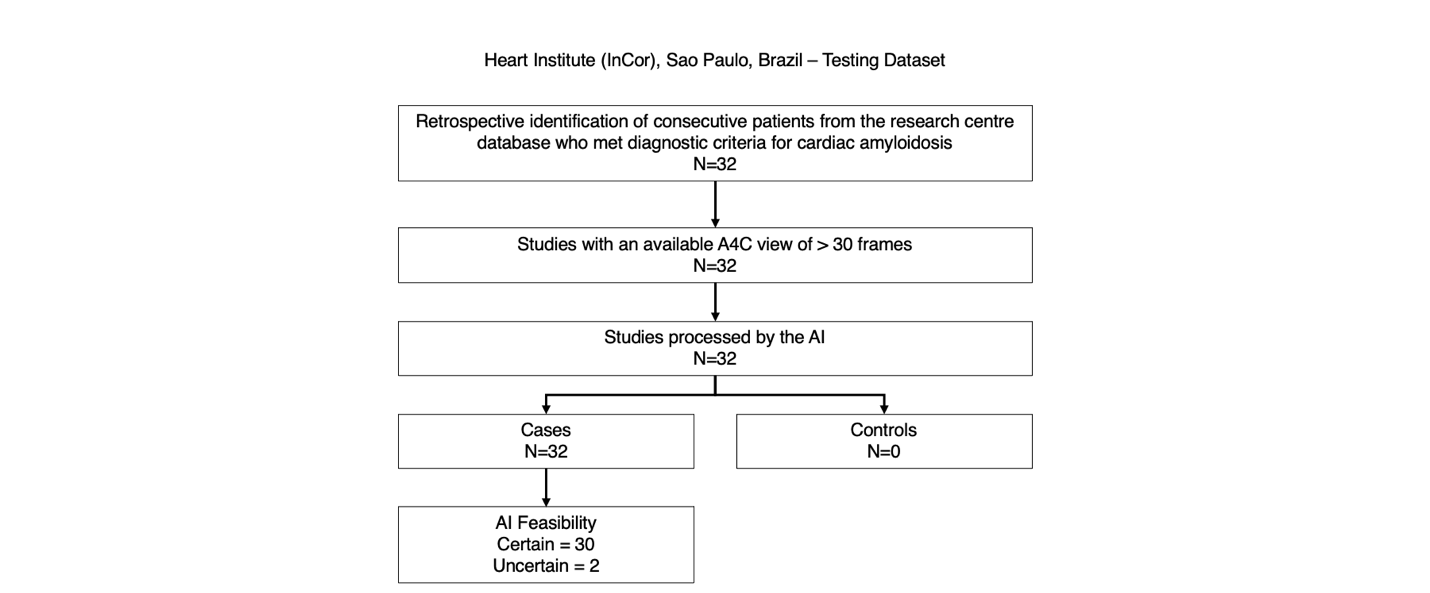


**Supplementary Figure 11.** Identification of patients from Heart Institute (InCor), Sao Paulo, Brazil for external validation and subsequent flow of data through the study.


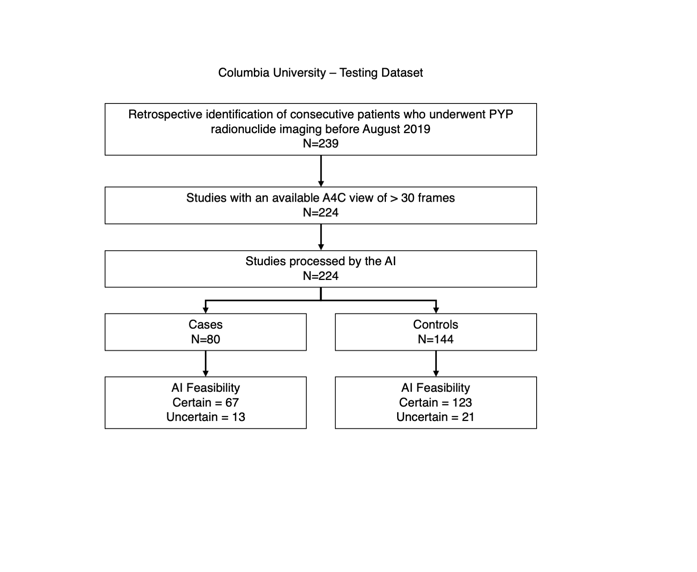


**Supplementary Figure 12.** Identification of patients from Columbia University for external validation and subsequent flow of data through the study.


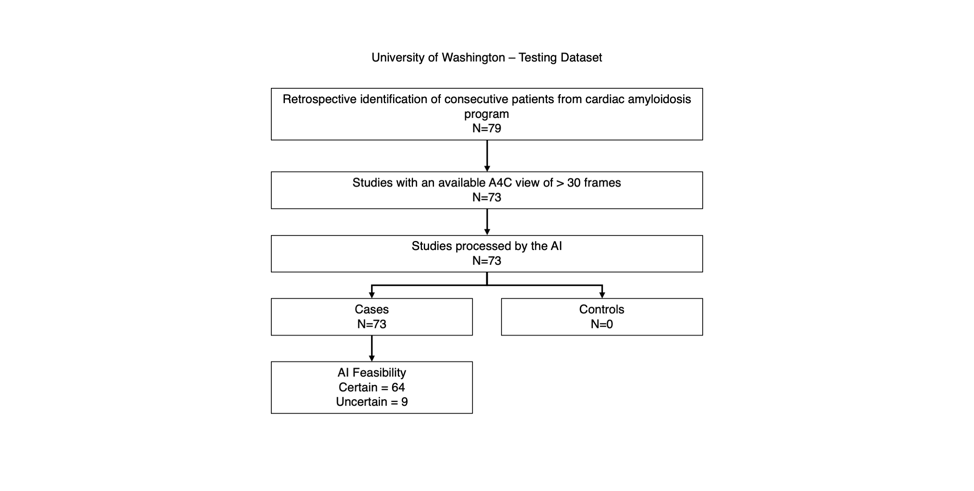


**Supplementary Figure 13.** Identification of patients from the University of Washington for external validation and subsequent flow of data through the study.


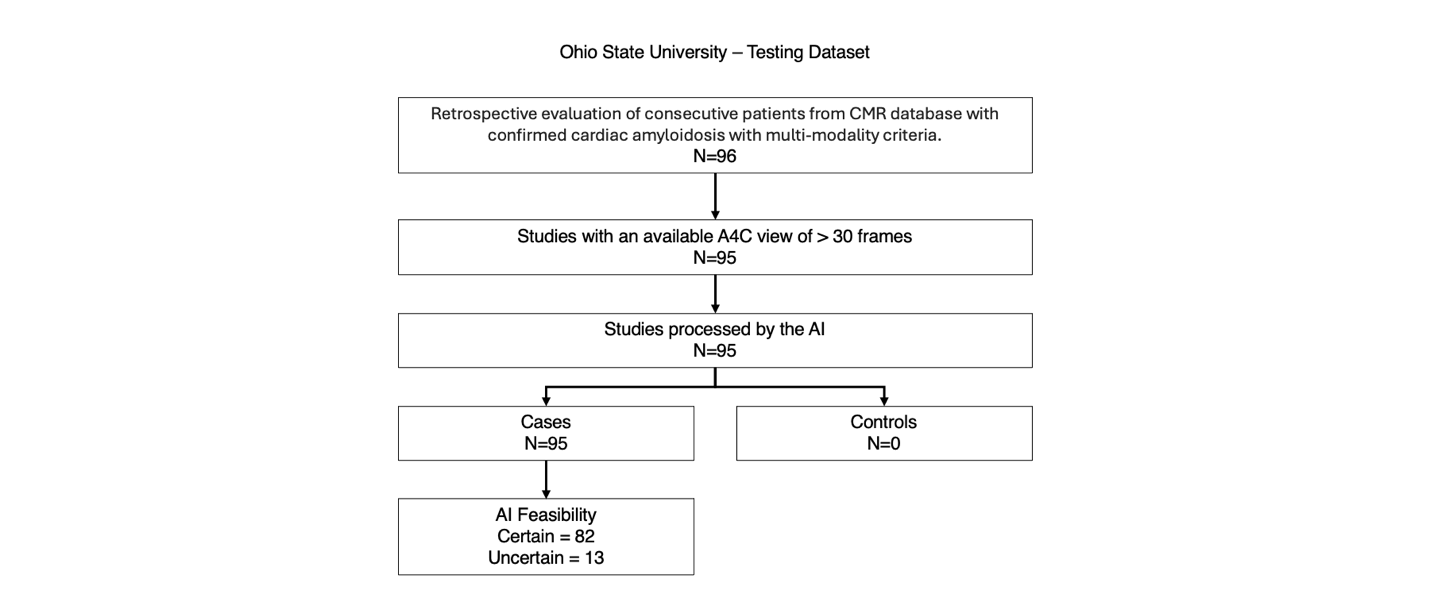


**Supplementary Figure 14.** Identification of patients from Ohio State University for external validation and subsequent flow of data through the study.


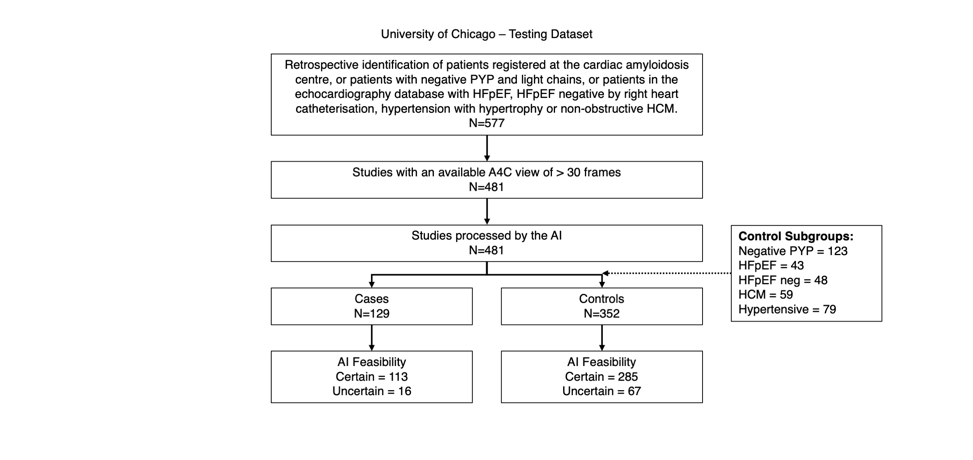
 **Supplementary Figure 15.** Identification of patients from the University of Chicago for external validation and subsequent flow of data through the study.


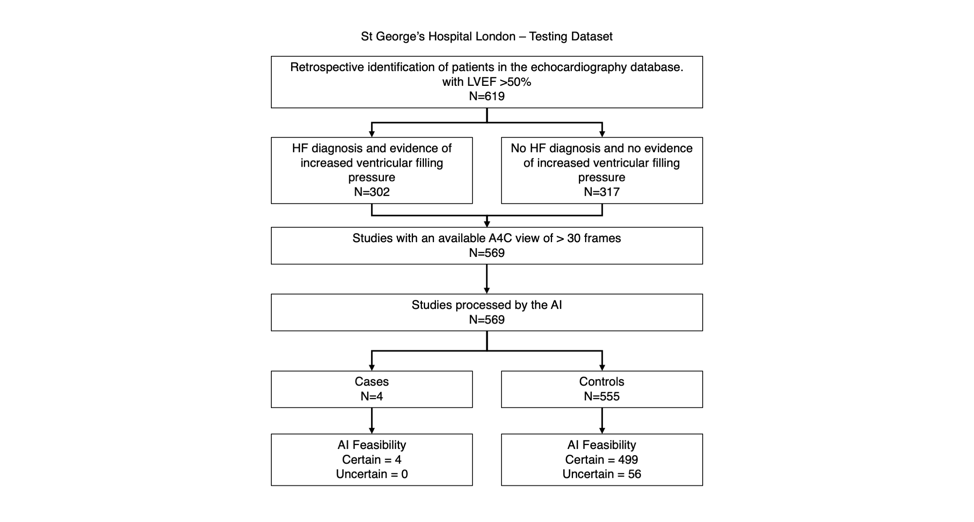


**Supplementary Figure 16.** Identification of patients from St George’s University Hospital, London for external validation and subsequent flow of data through the study.


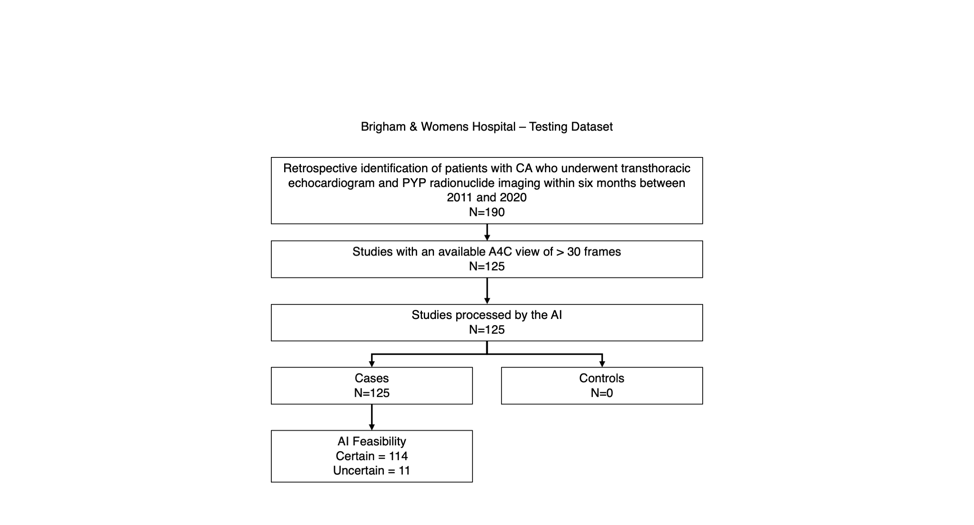


**Supplementary Figure 17.** Identification of patients from Brigham & Women’s Hospital for external validation and subsequent flow of data through the study.


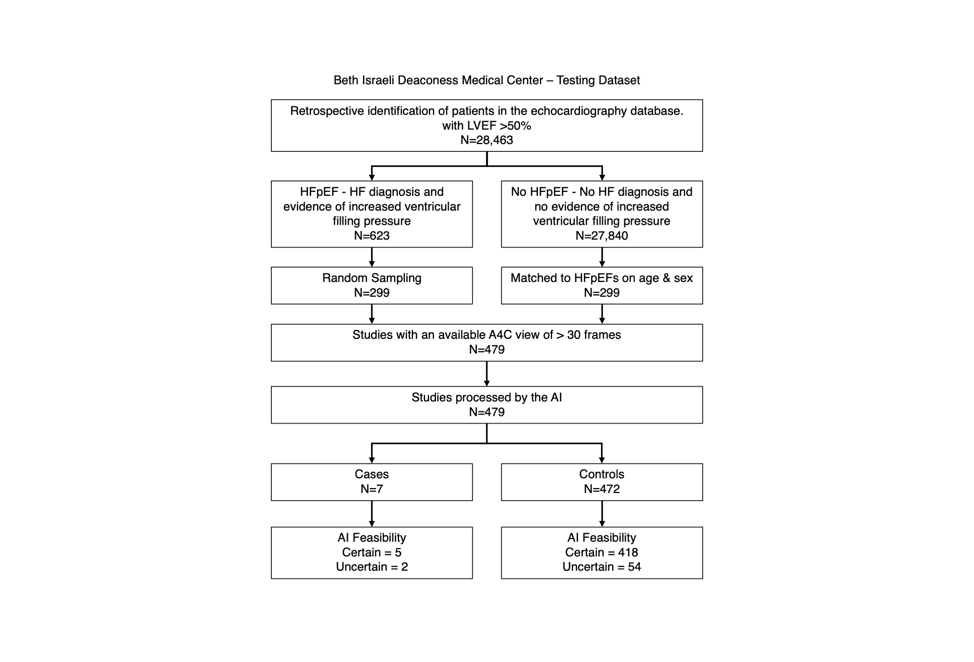


**Supplementary Figure 18.** Identification of patients from Beth Israel Deaconess Medical Center for external validation and subsequent flow of data through the study.


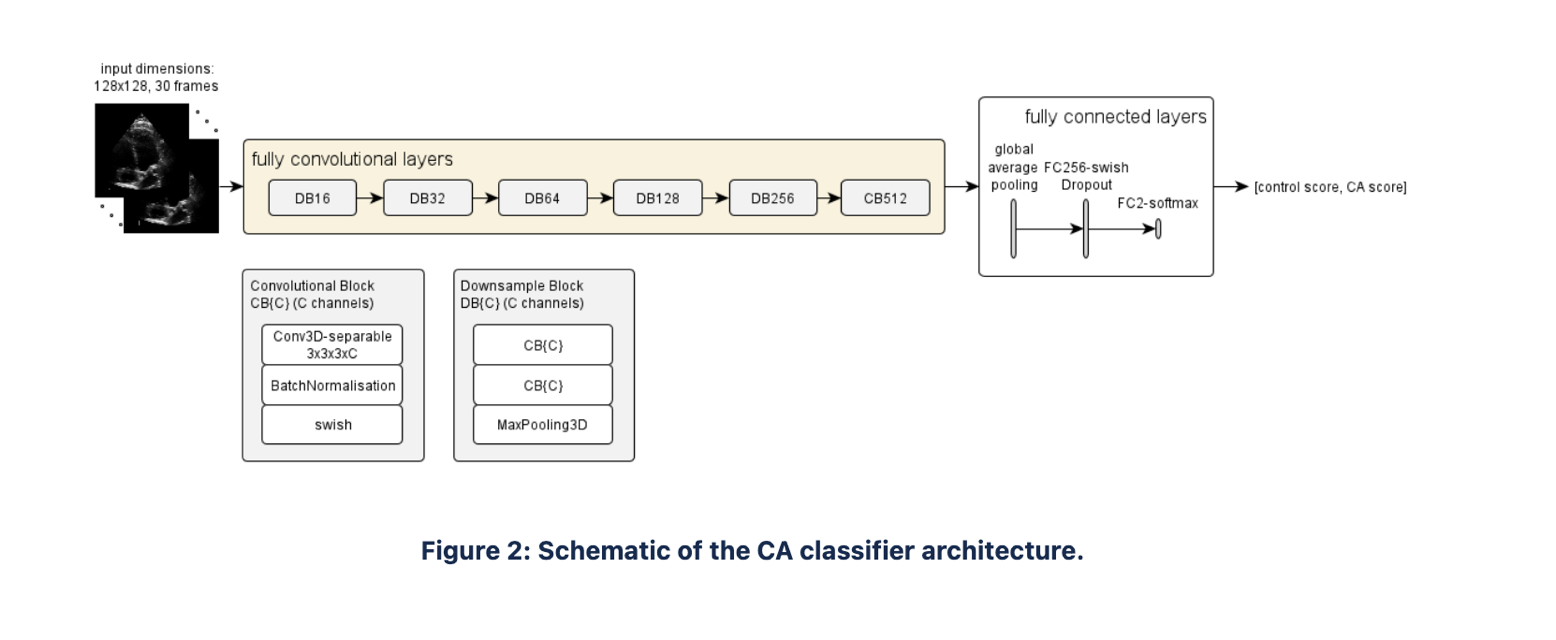


**Supplementary Figure 19.** Schematic of the AI model architecture.

**Supplementary Table 10**: Descriptive statistics of the external validation cohort and subgroups

|  | Cases | | | Controls | | |
| --- | --- | --- | --- | --- | --- | --- |
|  | Entire External Validation  n= 597 | Tc-PyP Referral Subgroup  n= 80 | Matched Subgroup  n= 285 | Entire External Validation  n=2,122 | Tc-PyP Referral Subgroup  n= 267 | Matched Subgroup  n= 285 |
| Age, median(IQR) | 73. 65,79) [547]* | 78 (70,84) [80] | 73 (65,79.0) [285] | 71 (58.0,81.0) [1850] | 75.0 (66.24,82.0) [257] | 73.0 (65.0,79.0) [285] |
| Body mass index, kg/m2, median(IQR) | 25.93 (23.12,29.01) [428] | 27.12 (23.52,30.59) [80] | 25.64 (23.03,28.91) [220] | 28.28 (24.53,32.56) [1853] | 28.46 (24.57,32.4) [266] | 29.81 (25.35,34.96) [290] |
| Male n(%) | 470 (79.1) | 61 (76.2) | 252 (79.5) | 1054.0 (50.2) | 154 (57.7) | 252 (79.5) |
| Race, n(%) |  |  |  |  |  |  |
| White n(%) | 340 (58.3) | 15 (18.8) | 194 (61.6) | 1172 (63.6) | 122 (46.0) | 197 (70.1) |
| Black n(%) | 153 (26.2) | 45 (56.2) | 74 (23.5) | 369 (20.0) | 117 (44.2) | 63 (22.4) |
| Other race n(%) | 90 (15.4) | 20 (25.0) | 47 (14.9) | 303 (16.4) | 26 (9.8) | 21 (7.5) |
| Hypertension, n(%) | 233 (68.3) | 59 (73.8) | 108 (69.2) | 1385 (71.4) | 119 (82.6) | 211 (80.5) |
| Diabetes mellitus, n(%) | 80 (23.5) | 24 (30.0) | 36 (22.9) | 614 (31.4) | 52 (36.1) | 109 (41.4) |
| Prior Valve Surgery, n(%) | 4.0 (1.9) | 2.0 (2.5) | 1.0 (1.2) | 58.0 (4.8) | 19.0 (13.2) | 20.0 (9.4) |
| Ground Truth method, n(%) |  |  |  |  |  |  |
| Tc-PYP | 221.0 (46.5) | 1.0 (1.2) | 112.0 (44.6) | 258.0 (96.6) | 2.0 (0.7) | 76.0 (97.4) |
| EMB | 93.0 (19.6) | 14.0 (17.5) | 84.0 (33.5) | 2.0 (0.7) | 7.0 (2.6) | 1.0 (1.3) |
| ExCB | 161.0 (33.9) | 65.0 (81.2) | 55.0 (21.9) | 7.0 (2.6) | 258.0 (96.6) | 1.0 (1.3) |
| Left ventricular ejection fraction, (%), median(IQR) | 55.0 (46.58,62.4) [577] | 56.45 (47.0,64.0) [72] | 56.23 (47.38,64.1) [308] | 62.0 (56.0,67.0) [1835] | 54.0 (47.0,64.0) [252] | 60.0 (54.0,65.0) [283] |
| Interventricular septal thickness, mm, median(IQR) | 15.0 (13.0,17.0) [583] | 15.0 (14.0,17.0) [80] | 15.0 (12.4,17.0) [315] | 12.0 (10.0,14.0) [1598] | 15.0 (13.0,17.0) [263] | 13.0 (11.0,15.0) [283] |
| Left ventricular posterior wall thickness, mm, median(IQR) | 14.0 (12.0,16.2) [589] | 14.0 (13.0,16.0) [80] | 13.0 (11.0,14.0) [317] | 11.0 (9.0,13.0) [1929] | 14.0 (12.0,17.0) [260] | 13.0 (11.0,14.0) [317] |
| Left ventricular mass index, g/m2, median(IQR) | 128.69 (103.11,151.95) [425] | 134.29 (100.88,163.76) [80] | 122.63 (98.26,146.12) [219] | 106.0 (86.0,133.7) [869] | 128.17 (100.89,164.84) [262] | 123.0 (101.0,153.93) [232] |
| Ultrasound manufacturer, n(%) |  |  |  |  |  |  |
| Siemens | 35.0 (5.9) | 0.0 (0.0) | 23.0 (8.1) | 58.0 (3.7) | 0.0 (0.0) | 13.0 (4.9) |
| GE | 194.0 (32.8) | 0.0 (0.0) | 93.0 (32.9) | 784.0 (50.0) | 1.0 (0.4) | 103.0 (38.6) |
| Philips | 362.0 (61.3) | 80.0 (100.0) | 167.0 (59.0) | 725.0 (46.3) | 266.0 (99.6) | 151.0 (56.6) |

* The number of available data points are indicated in square brackets

EMB: Endomyocardial biopsy, ExCB: Extra-cardiac biopsy with cardiac imaging features typical for CA, PYP: technetium 99m pyrophosphate nuclear scintigraphy, IQR: Inter-quartile range.

**Supplementary Table 11**: Patient characteristics of cases and controls in the external validation dataset, split by the accuracy of the output of the AI model.

|  | Cases | | | Controls | | |
| --- | --- | --- | --- | --- | --- | --- |
| Variable | Correct  n=447 | Incorrect  n=79 | Uncertain  n=71 | Correct  n=1705 | Incorrect  n=125 | Uncertain  n=71 |
| Age, years, median(IQR) | 73.0 (65.0, 79.0) [406] | 71.0 (64.8, 80.0) [76] | 72.0 (64.0, 80.0) [65] | 70.0 (57.0, 80.0) [1514] | 72.0 (64.0, 83.0) [97] | 72.0 (58.0, 82.5) [239] |
| Body mass index, kg/m2, median(IQR) | 25.3 (22.8, 28.4) [317] | 27.8 (25.1, 32.5) [57] | 26.2 (24.0, 29.5) [54] | 28.4 (24.7, 32.7) [1516] | 25.6 (21.8, 31.0) [97] | 27.6 (23.9, 32.9) [240] |
| Male, n(%) | 363 (81.2) | 55 (71.4) | 52 (74.3) | 843 (50.0) | 68 (55.3) | 143 (49.1) |
| Black/African American, n(%) | 121 (27.6) | 16 (21.3) | 16 (22.9) | 241 (16.4) | 41 (37.6) | 87 (33.3) |
| White, n(%) | 253 (57.8) | 43 (57.3) | 44 (62.9) | 979 (66.4) | 53 (48.6) | 140 (53.6) |
| Other race, n (%) | 64 (14.6) | 16 (21.3) | 10 (14.3) | 254 (17.2) | 15 (13.8) | 34 (13.0) |
| Hypertension, n(%) | 170 (67.5) | 33 (68.8) | 30 (73.2) | 1107 (70.3) | 75 (72.1) | 203 (77.8) |
| Diabetes mellitus, n(%) | 58 (22.9) | 12 (25.0) | 10 (25.0) | 493 (30.9) | 30 (28.8) | 91 (35.4) |
| Prior valve surgery, n(%) | 2 (1.3) | 1 (3.6) | 1 (3.8) | 2 (1.3) | 1 (3.6) | 1 (3.8) |
| Left ventricular ejection fraction, (%), median(IQR) | 54.8 (46.0, 62.0) [432] | 55.2 (46.6, 62.9) [76] | 56.0 (47.4, 64.0) [69] | 62.0 (57.0, 67.0) [1503] | 59.5 (52.4, 64.8) [94] | 62.0 (56.0, 67.8) [238] |
| Interventricular septal thickness, mm, median(IQR) | 15.8 (13.0, 17.0) [440] | 13.0 (11.0, 15.4) [75] | 15.0 (13.0, 16.5) [68] | 11.0 (10.0, 14.0) [1264] | 14.0 (11.0, 16.0) [102] | 12.0 (10.0, 15.0) [232] |
| Left ventricular posterior wall thickness, mm, median(IQR) | 14.1 (13.0, 17.0) [443] | 12.0 (10.0, 14.0) [76] | 13.0 (11.0, 15.0) [70] | 10.0 (9.0, 12.0) [1572] | 12.5 (11.0, 14.0) [106] | 11.0 (10.0, 13.0) [251] |
| Left ventricular mass index, g/m2, median(IQR) | 129.9 (106.7, 153.3) [317] | 117.9 (84.5, 144.0) [56] | 132.2 (87.3, 151.9) [52] | 105.0 (85.5, 132.5) [678] | 128.3 (102.0, 148.7) [55] | 102.0 (84.1, 125.8) [136] |
| Ultrasound manufacturer, n(%) |  |  |  |  |  |  |
| GE | 153 (34.7) | 21 (26.6) | 20 (28.2) | 640 (51.9) | 37 (38.1) | 107 (45.3) |
| Philips | 260 (59.0) | 55 (69.6) | 47 (66.2) | 545 (44.2) | 57 (58.8) | 123 (52.1) |
| Siemens | 28 (6.3) | 3 (3.8) | 4 (5.6) | 49 (4.0) | 3 (3.1) | 6 (2.5) |
| Ground Truth Method, n(%) |  |  |  |  |  |  |
| EMB | 128 (35.2) | 15 (26.8) | 18 (32.7) | 5 (2.6) | 0 (0.0) | 2 (4.4) |
| ExCB | 69 (19.0) | 13 (23.2) | 11 (20.0) | 2 (1.0) | 0 (0.0) | 0 (0.0) |
| Tc-PYP | 167 (45.9) | 28 (50.0) | 26 (47.3) | 184 (96.3) | 31 (100.0) | 43 (95.6) |

*The number of available data points are indicated in square brackets

EMB: Endomyocardial biopsy, ExCB: Extra-cardiac biopsy with cardiac imaging features typical for CA, PYP: technetium 99m pyrophosphate nuclear scintigraphy, IQR: Inter-quartile range.


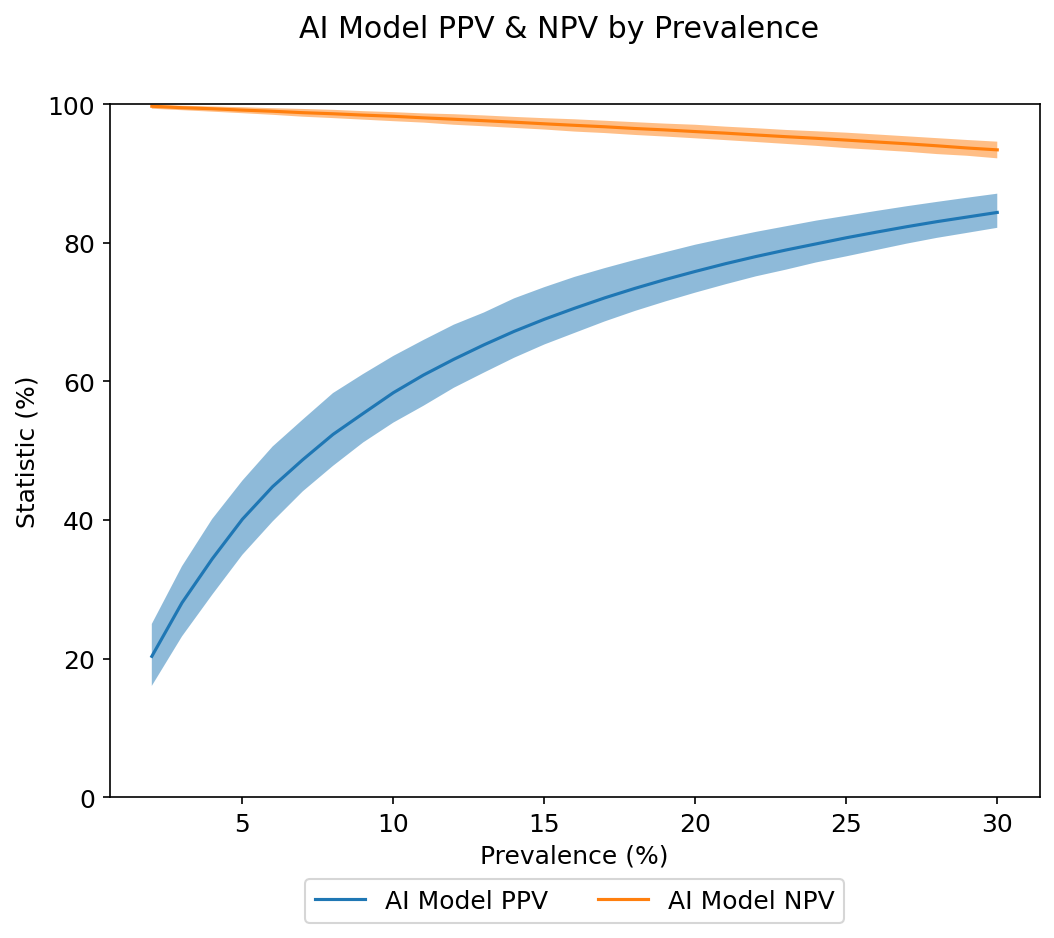


**Supplementary Figure 20:** PPV and NPV of the AI model at varying disease prevalence in the external testing dataset. Median values are represented by the solid line and shaded areas indicate the 2.5^th^ to 97.5^th^ percentiles, derived from bootstrapping.

**Supplementary Table 12:** Patient characteristics of the TCAS and IWT subset.

|  | Controls, n=237 | Cases, n=75 |
| --- | --- | --- |
| Age, median (IQR) | 79.0 (72.59,85.0) [237.0] | 78.0 (72.5,83.88) [75.0] |
| Body mass index, Kg/m2, median (IQR) | 29.69 (26.0,34.23) [237.0] | 26.76 (23.31,29.56) [75.0] |
| Male, n(%) | 147.0 (62.0) | 61.0 (81.3) |
| Black, n(%) | 11.0 (6.4) | 12.0 (16.0) |
| Other Race, n(%) | 18.0 (10.5) | 14.0 (18.7) |
| White, n(%) | 142.0 (83.0) | 49.0 (65.3) |
| Hypertension, n(%) | 212.0 (89.5) | 54.0 (72.0) |
| Diabetes mellitus, n(%) | 100.0 (42.2) | 22.0 (29.3) |
| Left ventricular ejection fraction, (%), median(IQR) | 60.0 (54.0,65.0) [237] | 57.0 (50.55,64.0) [75] |
| Interventricular septal thickness, mm, median(IQR) | 14.0 (12.0,16.0) [237] | 15.0 (14.0,17.0) [75] |
| Left ventricular posterior wall thickness, mm, median(IQR) | 13.0 (12.0,14.0) [237] | 14.0 (13.0,16.0) [75] |
| Left ventricular mass index, g/m2, median(IQR) | 127.0 (109.05,153.0) [237] | 133.29 (103.77,153.1) [75] |
| Ultrasound manufacturer |  |  |
| Siemens | 10.0 (4.2) | 0.0 (0.0) |
| GE | 73.0 (30.8) | 19.0 (25.3) |
| Philips | 154.0 (65.0) | 56.0 (74.7) |

The number of available data points are indicated in square brackets

**Supplementary Table 13:** Performance of the AI model, including uncertain predictions in tuning, external testing and all subgroups within the external testing datasets.

| Dataset | Sensitivity | Specificity | PPV | NPV |
| --- | --- | --- | --- | --- |
| Tuning | 90.2 (82.7, 96.3) | 85.4 (84.5, 86.2) | 7.7 (7.0, 8.3) | 99.8 (99.7, 99.9) |
| External Testing (all data) | 86.8 (83.6, 89.7) | 80.6 (78.8, 82.6) | 55.8 (53.5, 58.5) | 95.6 (94.6, 96.6) |
| External Testing (PYP Referrals) | 81.2 (71.4, 90.5) | 71.5 (65.7, 77.5) | 46.1 (40.0, 52.0) | 92.7 (89.4, 96.3) |
| External Testing (age, sex & wall thickness matched) | 86.3 (81.1, 90.8) | 77.5 (71.9, 82.9) | 79.4 (75.5, 83.4) | 85.0 (80.9, 89.4) |
| TCAS & IWT Subgroup | 80.0 (69.5, 89.8) | 92.4 (88.4, 95.8) | 76.9 (68.2, 86.0) | 93.6 (90.9, 96.6) |

**References**

1. Simonyan, K. & Zisserman, A. Very Deep Convolutional Networks for Large-Scale Image Recognition. 1–14 (2014) doi:10.1016/j.infsof.2008.09.005.

2. Akerman, A. P. *et al.* Automated Echocardiographic Detection of Heart Failure With Preserved Ejection Fraction Using Artificial Intelligence. *JACC: Advances* 100452 (2023) doi:10.1016/j.jacadv.2023.100452.

3. Chakravarti, N. Isotonic Median Regression: A Linear Programming Approach. *Mathematics of OR* **14**, 303–308 (1989).

4. Lindmark, K., Pilebro, B., Sundström, T. & Lindqvist, P. Prevalence of wild type transtyrethin cardiac amyloidosis in a heart failure clinic. *ESC Heart Failure* **8**, 745–749 (2021).

5. Aimo, A. *et al.* Redefining the epidemiology of cardiac amyloidosis. A systematic review and meta-analysis of screening studies. *European Journal of Heart Failure* **24**, 2342–2351 (2022).

6. Merlo, M. *et al.* Unmasking the prevalence of amyloid cardiomyopathy in the real world: results from Phase 2 of the AC-TIVE study, an Italian nationwide survey. *European Journal of Heart Failure* **24**, 1377–1386 (2022).

7. AbouEzzeddine, O. F. *et al.* Prevalence of Transthyretin Amyloid Cardiomyopathy in Heart Failure With Preserved Ejection Fraction. *JAMA Cardiology* **6**, 1267–1274 (2021).

8. Hendrycks, D. & Gimpel, K. A Baseline for Detecting Misclassified and Out-of-Distribution Examples in Neural Networks. Preprint at https://doi.org/10.48550/arXiv.1610.02136 (2018).

9. Kendall, A., Badrinarayanan, V. & Cipolla, R. Bayesian SegNet: Model Uncertainty in Deep Convolutional Encoder-Decoder Architectures for Scene Understanding. Preprint at https://doi.org/10.48550/arXiv.1511.02680 (2016).

10. Akerman, A. P. *et al.* Automated Echocardiographic Detection of Heart Failure With Preserved Ejection Fraction Using Artificial Intelligence. *JACC: Advances* **2**, 100452 (2023).

11. Dorbala, S. *et al.* ASNC/AHA/ASE/EANM/HFSA/ISA/SCMR/SNMMI expert consensus recommendations for multimodality imaging in cardiac amyloidosis: Part 2 of 2—Diagnostic criteria and appropriate utilization. *J. Nucl. Cardiol.* **27**, 659–673 (2020).

12. Steinberg, D. M., Fine, J. & Chappell, R. Sample size for positive and negative predictive value in diagnostic research using case–control designs. *Biostatistics* **10**, 94–105 (2009).

13. Arana-Achaga, X. *et al.* Development and Validation of a Prediction Model and Score for Transthyretin Cardiac Amyloidosis Diagnosis: T-Amylo. *JACC: Cardiovascular Imaging* (2023) doi:10.1016/j.jcmg.2023.05.002.

14. Boldrini, M. *et al.* Multiparametric Echocardiography Scores for the Diagnosis of Cardiac Amyloidosis. *JACC: Cardiovascular Imaging* **13**, 909–920 (2020).

15. Cuddy, S. A. *et al.* Optimal Echocardiographic Parameters to Improve the Diagnostic Yield of Tc-99m-Bone Avid Tracer Cardiac Scintigraphy for Transthyretin Cardiac Amyloidosis. *Circ: Cardiovascular Imaging* **15**, (2022).

16. Kyrouac, D. *et al.* Echocardiographic and clinical predictors of cardiac amyloidosis: limitations of apical sparing. *ESC Heart Fail* **9**, 385–397 (2022).

17. Löfbacka, V. *et al.* Combining ECG and echocardiography to identify transthyretin cardiac amyloidosis in heart failure. *Clinical Physiology and Functional Imaging* **41**, 408–416 (2021).

18. Nicol, M. *et al.* Diagnostic score of cardiac involvement in AL amyloidosis. *European heart journal cardiovascular Imaging* **21**, 542–548 (2020).

19. Pagourelias, E. D. *et al.* Echo Parameters for Differential Diagnosis in Cardiac Amyloidosis. *Circulation: Cardiovascular Imaging* **10**, e005588 (2017).

20. Phelan, D. *et al.* Relative apical sparing of longitudinal strain using two-dimensional speckle-tracking echocardiography is both sensitive and specific for the diagnosis of cardiac amyloidosis. *Heart* **98**, 1442–1448 (2012).

21. Robin, G. *et al.* Value of Longitudinal Strain to Identify Wild-Type Transthyretin Amyloidosis in Patients With Aortic Stenosis. *Circ J* **85**, 1494–1504 (2021).

22. Schiano-Lomoriello, V. *et al.* Longitudinal strain of left ventricular basal segments and E/e′ ratio differentiate primary cardiac amyloidosis at presentation from hypertensive hypertrophy: an automated function imaging study. *Echocardiography* **33**, 1335–1343 (2016).

23. Usuku, H. *et al.* Usefulness of automatic assessment for longitudinal strain to diagnose wild-type transthyretin amyloid cardiomyopathy. *IJC Heart & Vasculature* **47**, 101227 (2023).
